# Genetic and Phenotypic Features of a Novel Acinetobacter Species, Strain A47, Isolated From the Clinical Setting

**DOI:** 10.3389/fmicb.2019.01375

**Published:** 2019-06-18

**Authors:** Sareda T. J. Schramm, Kori Place, Sabrina Montaña, Marisa Almuzara, Sammie Fung, Jennifer S. Fernandez, Marisel R. Tuttobene, Adrián Golic, Matías Altilio, German M. Traglia, Carlos Vay, Maria Alejandra Mussi, Andres Iriarte, Maria Soledad Ramirez

**Affiliations:** ^1^Department of Biological Science, California State University Fullerton, Fullerton, CA, United States; ^2^Facultad de Medicina, Instituto de Microbiología y Parasitología Médica (IMPaM, UBA-CONICET), Universidad de Buenos Aires, Buenos Aires, Argentina; ^3^Laboratorio de Bacteriología Clínica, Departamento de Bioquímica Clínica, Hosp. de Clínicas José de San Martín, Facultad de Farmacia y Bioquímica, Universidad de Buenos Aires, Buenos Aires, Argentina; ^4^Centro de Estudios Fotosintéticos y Bioquímicos (CEFOBI – CONICET), Rosario, Argentina; ^5^Laboratorio de Biología Computacional, Departamento de Desarrollo Biotecnológico, Instituto de Higiene, Facultad de Medicina, Universidad de la República, Montevideo, Uruguay

**Keywords:** *Acinetobacter* spp., biofilm, antibiotic resistance, virulence traits, biofilm, natural transformation

## Abstract

In 2014, a novel species of *Acinetobacter*, strain A47, determined to be hospital-acquired was recovered from a single patient soft tissue sample following a traumatic accident. The complexity of the *Acinetobacter* genus has been established, and every year novel species are identified. However, specific features and virulence factors that allow members of this genus to be successful pathogens are not well understood. Utilizing both genomic and phenotypic approaches, we identified distinct features and potential virulence factors of the A47 strain to understand its pathobiology. *In silico* analyses confirmed the uniqueness of this strain and other comparative and sequence analyses were used to study the evolution of relevant features identified in this isolate. The A47 genome was further analyzed for genes associated with virulence and genes involved in type IV pili (T4P) biogenesis, hemolysis, type VI secretion system (T6SS), and novel antibiotic resistance determinants were identified. A47 exhibited natural transformation with both genomic and plasmid DNA. It was able to form biofilms on different surfaces, to cause hemolysis of sheep and rabbit erythrocytes, and to kill competitor bacteria. Additionally, surface structures with non-uniform length were visualized with scanning electron microscopy and proposed as pili-like structures. Furthermore, the A47 genome revealed the presence of two putative BLUF type photoreceptors, and phenotypic assays confirmed the modulation by light of different virulence traits. Taken together, these results provide insight into the pathobiology of A47, which exhibits multiple virulence factors, natural transformation, and the ability to sense and respond to light, which may contribute to the success of an A47 as a hospital dwelling pathogen.

## Introduction

The genus *Acinetobacter* represents an important group of pathogens. Currently, there are 52 species of *Acinetobacter* with assigned names^1^. However, the majority of research focuses on *Acinetobacter baumannii*, the most frequent cause of hospital-acquired infections (Turton et al., 2010; Lee et al., 2011; Weber et al., 2016). Recently with the development of other diagnostic tools and technological advancements, other members of the *Acinetobacter* genus have also been identified as causative agents of hospital-acquired infections (Turton et al., 2010; Karah et al., 2011). Although *A. baumannii* is still the most significant and common nosocomial pathogen, additional *Acinetobacter* species are gaining in clinical relevance.

The extreme genome plasticity and the ability to acquire foreign DNA has played an essential role in making some species of *Acinetobacter* successful pathogens. Horizontal gene transfer (HGT) allows bacteria to acquire and share DNA through different processes (conjugation, transduction, and transformation). Natural transformation is not fully understood, but its relevance in the spread of antibiotic resistance is unprecedented. Several species of *Acinetobacter* have been documented to naturally acquire foreign DNA (Palmen et al., 1993; Traglia et al., 2014). Transmembrane type IV pili (T4P) represent an important mechanism for acquiring exogenous DNA from the environment (Fronzes et al., 2008). T4P, which are complex structures composed of many proteins, is implicated in both the acquisition of exogenous DNA and the ability to overcome repulsive electrostatic forces during bacterial attachment to surfaces (Giltner et al., 2012; Berne et al., 2015). Initial attachment is imperative for the formation of complex biofilms, allowing the bacteria to survive antimicrobial treatment and maintain virulence. Biofilms protect the associated bacteria by decreasing the diffusion of some antibiotics or rendering them inactive before they can reach a subcellular target (Anderl et al., 2000). Additionally, some susceptible bacteria can tolerate antibiotics within the biofilm due to recalcitrance of biofilm bacteria toward antibiotics (Lebeaux et al., 2014). Biofilms are a unique virulence factor which provides bacteria with the ability to survive or tolerate antimicrobial treatment. It was previously recognized that *A. baumannii* perceives and responds to light modulating global features of its physiology through the BlsA photoreceptor encoded in its genome (Mussi et al., 2010). Light can modulate motility, biofilm formation, and virulence against *Candida albicans* in *A. baumannii* (Mussi et al., 2010). Furthermore, light regulates metabolic pathways, susceptibility and tolerance to some antibiotics, antioxidant enzyme levels such as catalase, likely contributing to bacterial persistence in adverse environments (Ramirez et al., 2015; Muller et al., 2017).

Extensive genome variation has generated interesting phenotypic variations throughout the *Acinetobacter* genus. One example of his is hemolytic activity, which not only varies by species but also between isolates (Tayabali et al., 2012; Dahdouh et al., 2016). Studies have identified all three types of hemolytic activity (α, β, and γ) in *Acinetobacter*, being the most uncommon the β-hemolysis (Tayabali et al., 2012; Dahdouh et al., 2016).

A taxonomically unique strain, *Acinetobacter* strain A47 (referred to as A47) recovered from a single patient soft tissue samples following a traumatic accident in 2014 was previously described (Almuzara et al., 2015; Traglia et al., 2015).

In this study, using both genomic and phenotypic approach, we aimed to characterize important mechanisms which may influence the pathobiology of A47 infections. *In silico* analyses revealed the phylogenetic position of this isolate among closely related strains and showed the uniqueness of it. The analysis of the A47 genome identified all T4P genes, and associated phenotypes with a functional T4P, such as natural competence and biofilm formation, were confirmed. Additionally, A47 was shown to modulate different virulence traits under blue light. Further genetic analyses led to the identification of multiple virulence factors, including genes associated with hemolysis and antibiotic resistance determinants. Although A47 was found to be susceptible to ampicillin-sulbactam, piperacillin-tazobactam, ceftazidime, cefepime, imipenem, meropenem, amikacin, gentamicin, ciprofloxacin, colistin, and trimethoprim-sulfamethoxazole (Almuzara et al., 2015; Traglia et al., 2015), the potential for this species to become a more significant threat should not be ignored, as previous studies have demonstrated antibiotic resistance and virulence are not consistently correlated (Tayabali et al., 2012; Giannouli et al., 2013). A47 harbors virulence factors which may cause additional problems concerning treatment options and pathobiology. As this is the initial isolation of A47, identifying mechanisms of virulence at a genetic and phenotypic level adds not only to the understanding of this novel *Acinetobacter* species but also to the growing knowledge of the *Acinetobacter* genus.

## Materials and Methods

### Bacterial Strains and Plasmids

*Acinetobacter* strain A47, a taxonomically unique species recovered from a single patient soft tissue sample following a traumatic accident, was used in the present work (Almuzara et al., 2015). Moreover, *A. baumannii* strains ATCC 17978, A144, Ab33405, and *A. haemolyticus* strain A23 were also used (Vilacoba et al., 2013). Plasmids pDSRed (4.5 kbp) and pJHCMW1 (11.3 kbp) which harbor a Kan^R^ gene were extracted from *Escherichia coli* TOP10 cells using QIAprep Spin Miniprep Kit following manufacturer protocol (Qiagen Germantown, MD, United States) and used for transformation assays.

### Whole Genome Sequence

The genome of A47 was previously sequenced using Illumina MiSeq at the Argentinian Consortium of Genomic Technology and reported in Traglia et al. (2015). Open reading frame (ORF) prediction was previously performed using the RAST server (Aziz et al., 2008). SEED source genome annotations identified known genes in the A47 genome under default parameters (Overbeek et al., 2014). Gene annotation was confirmed by BLAST (version 2.0) software at NCBI^2^.

### Homologous Gene Clustering, Phylogenetic Analysis, and Average Nucleotide Identity

The phylogenetic position of A47 within *Acinetobacter* genus was previously defined (Traglia et al., 2015). In order to get a more detailed phylogenetic position of A47 a group of closely related genomes to A47 strain was defined based on this previous published result. Then, a phylogenetic analysis of this group was done. The closely related genome group used for comparative analyses comprises 55 strains plus A47 (Supplementary Table S1). Homologous gene families among the 56 analyzed genomes were identified using the OrthoMCL method (Li et al., 2003) implemented in the get_homologous software package, version 07082017 (Contreras-Moreira and Vinuesa, 2013). Blastp search minimums for e-value, identity and query coverage were 1 × 10^–5^, 30 and 75%, respectively. Thousand three hundred and eighty-three clusters of putative orthologous sequences were identified among the analyzed genomes. Clustal Omega v1.2.0 was used to align protein sequences (Sievers et al., 2011). Gblock with default parameters was used to trim low quality aligned regions (Castresana, 2000).

Two different strategies were used for phylogenetic reconstruction: (1) PHYML version 3.1 was used to generate a maximum-likelihood phylogenetic tree for each alignment of orthologous protein sequences, using five random starting trees. Sumtrees.py script was then used to generate a consensus tree from the 1383 generated phylograms (Sukumaran and Holder, 2010). (2) The alignments of the 1383 orthologous proteins were concatenated. Then, FastTree version 2.1 was used for building an approximately maximum-likelihood phylogenetic tree. In both cases, consensus and concatenated approaches, the amino acid LG + G model were used with eight categories (Guindon and Gascuel, 2003). Branch supports were evaluated using the SH-like test (Guindon et al., 2010; Supplementary Figure S2).

The average nucleotide identity (ANI) score between A47 and other closely related genomes were estimated. The ANI is used to delineate species using genome sequence data. Two genomes displaying an ANI value of 95% or higher are considered to be the same species (Goris et al., 2007). Two-way ANI (reciprocal best hits based comparison) was estimated by the ani.rb script, developed by Luis M. Rodriguez-R and available at enveomics.blogspot.com.

### Comparative Genomic Analysis, Gene Content, and Sequence Analysis

Comparative genome analysis was performed with the open-source MAUVE aligner version 2.3.1 (Darling et al., 2004). ARG-ANNOT and ISfinder softwares were used to identify antibiotic resistance genes and insertion sequences, respectively (Siguier et al., 2006; Gupta et al., 2014). Phage and prophage sequences were identified using PHAST (Zhou et al., 2011). PlasmidFinder was used to detect the presence of *Enterobacteriaceae* plasmids (Carattoli et al., 2014). In addition, CRISPRCASFinder and RASTA-Bacteria software were used to searched for the CRISPR-Cas and toxin-antitoxin systems, respectively (Sevin and Barloy-Hubler, 2007; Couvin et al., 2018).

The group of unique genes of strain A47 was defined based on the get_homologous result, see above.

### Molecular Evolutionary Studies of *bla*_OXA–like_ Genes and Phylogenetic Distribution of Hemolytic Activity-Related Genes

Homologous sequences of the *bla*_OXA–like_ gene and putative hemolytic related genes found in A47 were identified in closely related genomes using the BLASTP tool. A minimum identity value of 40% and a minimum coverage of 75% were set as thresholds for positive hits. *bla*_OXA–like_ homologous proteins were aligned using Clustal Omega v1.2.0. Then, a phylogeny was build using PHYML v3.1 as mention above (Sukumaran and Holder, 2010). Amino acid pairwise *p*-distance was estimated in MEGA version 7.0 (Kumar et al., 2016). Aligned proteins were back-translated to the known DNA sequences by means of the tranalign program from the EMBOSS package (Rice et al., 2000) and visualized using Bioedit v7.5 (Hall, 2011). Site and branch models dN/dS selection tests were done in the Datamonkey web server (Weaver et al., 2018), Fixed Effects Likelihood test and adaptive Branch Site REL tests (Kosakovsky Pond and Frost, 2005; Smith et al., 2015), respectively. The ratio of non-synonymous changes per non-synonymous sites (dN) over synonymous changes per synonymous sites (dS) is used to identify sites or lineages that evolve under negative, neutral or positive selection regimes. Hemolytic related genes were mapped in the phylogeny of the group according to the BLAST results, using the iTOL web server (Letunic and Bork, 2016).

### Transformation Assays

To perform the transformation assays, two KAN^R^ plasmids, pJHCMW1 (11.3 kbp) or pDSRed (4.5 kbp) (Sarno et al., 2002; Traglia et al., 2016), were used. *A. baumannii* A144 and Ab33405 (GenBank accession numbers JQSF01000000 and JPXZ00000000, respectively) strains, known to harbor many genes conferring resistance to aminoglycosides (Vilacoba et al., 2013), were used as the source of total genomic DNA (gDNA).

Transformation assays were carried out as previously described (Ramirez et al., 2010). Briefly, late-stationary-phase cells (OD_600_:1) of A47 were mixed with either donor gDNA or plasmid DNA, incubated for 1h at 37°C, and then plated on LB agar supplemented with 10 μg/ml KAN overnight at 37°C. Following incubation, KAN^R^ colonies representing individual transformation events were counted. An average of 15 colonies (transformants) were checked and confirmed in every independent transformation experiment by PCR reactions targeting the genes *aac(6′)-Ib*, *aac(3)-IIa*, and *aph(3′)-Ia*. Total colony forming units (CFUs) were quantified by plating dilutions of non-transformed A47 cells on LB agar and incubated at 37°C overnight. The calculated transformation frequency represented the number of KAN^R^ colonies per CFU. Experiments were repeated three times (*n* = 3) and statistical significance was determined using ANOVA.

Antimicrobial susceptibility testing was performed to assess susceptibility levels of selected transformants cells. Disk diffusion was used to assess susceptibility levels to amikacin (AK), ceftazidime (CAZ), ciprofloxacin (CIP), gentamicin (GN), cefepime (FEP), imipenem (IMP), meropenem (MEM), minocycline (MH), trimethoprim/sulfamethoxazole (SXT), tetracycline (TE), and tigecycline (TGC) disks (Oxoid™, Basingstoke, United Kingdom) according to the Clinical and Laboratory Standards Institute (Clinical and Laboratory Standards Institute, 2017). Minimum Inhibitory Concentration (MIC) to AK, CAZ, GN, and cefotaxime (CT) were measured using commercial strips (bioMérieux, Marcy-l’Etoile, FR) following the gradient diffusion method (E-test method) as recommended by the supplier (Jorgensen and Ferraro, 2009).

### Biofilm Formation

Quantification of biofilm production, in glass and polystyrene tube, was carried out using a protocol adapted from previously described methods (O’toole and Kolter, 1998; Tomaras et al., 2003; Mussi et al., 2010). Quantification of biofilm was reported as a ratio of biofilm to total biomass (OD_580_/OD_600_) for three independent experiments (*n* = 3). By reporting biofilm as a ratio of total biomass, each value is normalized to the total biomass to account for any variation in growth due to the different abiotic tubes. *A. baumannii* strain ATCC 17978 was used as a control (Eijkelkamp et al., 2011; Nait Chabane et al., 2014). Additionally, by normalizing to total cell mass, differences due to the growth rates of A47 and ATCC 17978 do not obscure reported biofilm formation. Statistical significance was determined using a two-tailed Student’s *t*-test and one-way ANOVA using GraphPad Prism (GraphPad Software, San Diego, CA, United States).

### Scanning Electron Microscopy

The surface of A47 was visualized using scanning electron microscopy (SEM). A working protein fixing solution (2% formaldehyde, 2% glutaraldehyde, and 0.1 M phosphate buffer) was used to fix A47 cells. A section of A47 was extracted and transferred into a sterile glass vial. Lipids and fatty acids were fixed with 2% osmium tetroxide. The sample was dehydrated with ethanol and mounted to a degreased stub using double stick tape. The stub was coated in gold/platinum and imaged with the Hitachi S-2400 scanning electron microscope.

### Hemolysis Assays

Hemolytic activity was determined using a previously described protocol with minor changes (Tayabali et al., 2012). Tryptic soy agar (TSA) plates supplemented with 5% sheep’s blood (blood agar plates; Hardy Diagnostics, Santa Maria, CA, United States) were inoculated by transferring colonies of A47 to a blood agar plate. Blood agar plates were incubated for up to 72 h at 37°C. At 24, 48, and 72 h post-inoculation the diameter of the colony (D_1_) and the diameter of the clearing (D_2_) was measured and recorded in mm. Hemolysis was quantified by calculating the ratio of the diameter of the clearing to the diameter of the colony (D_2_/D_1_). Independent biological samples were used for every trial (*n* = 16). *A. haemolyticus* strain A23 strain was used for comparison.

An additional assay was conducted to measure hemolytic activity using defibrinated rabbit erythrocytes, following a previously described protocol with modifications (Ramsey et al., 2016). Individual colonies of A47 were cultured in 5 mL LB broth overnight with agitation, then diluted 1/100 with LB broth. Defibrinated rabbit erythrocytes (Hemostat Laboratories, Dixon, CA, United States) were diluted to 10% (v/v) in 1X phosphate buffered saline (PBS). 60 μL of 1/100 A47 cultures and 140 μL of 10% rabbit blood were combined in wells of a flat-bottom 96-well plate and incubated for 12 h at 37°C. The plate was then centrifuged for 10 min at 3000 rpm, 100 μL of the supernatant was transferred to a new well; absorbance of the supernatant was measured at 540 and 630 nm. Independent biological samples were used for every trial (*n* = 4) and performed in quadruplicates. LB without bacteria served as a negative control, as well as a normalization for hemolysis.

### Blue Light Treatments

Blue light treatments were performed as described in previous studies (Mussi et al., 2010; Golic et al., 2013; Ramirez et al., 2015). Cells were incubated for 26 h (or else specified) at 24°C in the dark or under blue light emitted by nine-LED (light-emitting diode) arrays with an intensity of 6–10 μmol photons/m^2^/s. Each array was built using three-LED module strips emitting blue light with emission peaks centered at 462 nm determined using a LI-COR LI-1800 spectroradiometer (Mussi et al., 2010).

### Growth of A47 on Phenylacetic Acid

To test the ability of A47 to grow on phenylacetic acid (PAA), 1/100 dilutions of overnight cultures grown in LB Difco were washed with PBS 1X and inoculated in either M9 solid medium supplemented with 5 or 10 mM PAA or in LB Difco medium (Fisher Scientific) and incubated at 24°C under blue light or in the dark. The experiments were performed in triplicates.

### Quantification of Trehalose

The strain was grown in LB Difco medium until it reached an OD660 of 0.4 under blue light or in the dark, and sugars were extracted from bacterial cells as described in Zeidler et al. (2017). Trehalose content was quantified as previously described by measuring glucose increments after trehalase (Sigma) treatment (Muller et al., 2017). Glucose calibration curves were generated using glucose (Sigma) as standards and glucose was measured using a glucose oxidase kit (Glicemia enzimaìtica AA liìquida) under the recommendations of the manufacturer (Wiener, Argentina) before and after trehalase treatment. The experiments were performed in triplicates.

### Cell Motility

Cell motility was tested on “swimming agarose” (Tryptone 1%, NaCl 0.5%, agarose 0.3%; 5) or LB agarose (Peptone 1%, NaCl 1%, yeast extract 0.5%, agarose 0.3%) plates incubated in the presence or absence of light, as described previously (Mussi et al., 2010; Golic et al., 2013). All assays were performed in triplicates for both light and dark conditions.

## Results

### A47 Genome Features and Comparative Genomic Analysis

**FIGURE 1:**
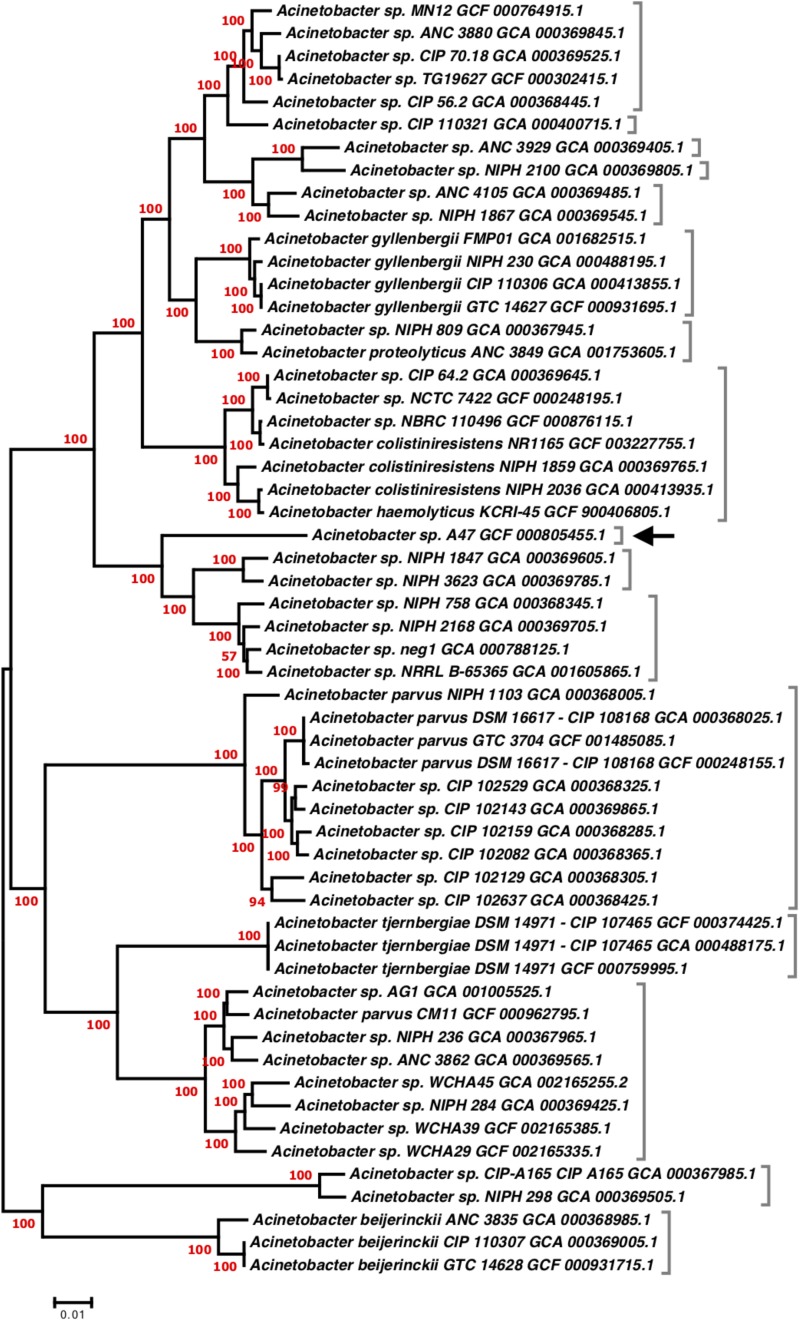
Approximate maximum likelihood phylogenetic tree of A47 and closely related assemblies. The phylogenetic tree was build based on 1383 concatenated orthologous proteins. The tree was inferred using FastTree version 2.1, with LG + G model. The SH-like test was used to evaluate branch supports and indicated as red values next to nodes. Genomes from the same species based on two-way ANI score (>95%) were indicated with brackets. The position of A47 is indicated with a black arrow.

The genome of A47 is 3,915,593 bp with a corresponding G+C content of 44.5% (Traglia et al., 2015). RAST annotation software predicted a total of 3627 ORFs, of which 281 are unique genes, which are defined as genes with no homologs in any closely related genome. These unique genes are distributed in at least 28 clusters (Supplementary Table S2). Almost 70% of the identified singletons are hypothetical proteins; and other unique genes coding for transporters, phage-related proteins, transcriptional regulators, among other functional categories. ANI values support the previous report that A47 does not belong to any hitherto known taxa and represents a unique species of *Acinetobacter* (Supplementary Table S3). Phylogenetic analysis using 56 publicly available genomes identified the genetic relationship between A47 and closely related strains. Two robust phylogenetic trees were built based on the sequences of 1383 aligned orthologs. Implemented strategies showed highly similar branching pattern; however, the phylogenetic tree generated with FastTree was built based on concatenated sequences and displayed higher node support values (Figure 1). A47 grouped with strong statistical support as a monophyletic cluster with two lineages that also represent new species (node support = 100) (see ANI results in Supplementary Table S3). Then, as a sister group of this monophyletic cluster, three equally distant and defined species were found: *A. gyllenbergii*, *A. colistiniresistens*, and *A. proteolyticus*.

### Genetic Analysis of Virulence Genes Associated With Distinct Phenotypes

Initial phenotypic testing of A47 indicated it was β-hemolytic (Almuzara et al., 2015), leading to the search for putative genes related with hemolytic activity. Thus, four protein-coding genes were identified in the A47 genome including a putative hemolysin (WP_038344358.1) with 85% identity to a previously described protein in *A. haemolyticus* TJS01 (Accession number: APR70514.1), a putative hemolysin translocator protein, HylD (WP_038343297.1) with 97% identity to a protein in *Acinetobacter* sp. WC-323, a predicted membrane protein (WP_038343513.1) with 78% identity to hemolysin III, Hly III, present in *A. junni* 65 (Accession number: CP019041.1), and ahemolysin activation/secretion family protein, HecB, (WP_052209140.1) with 77% identity to a protein identified in *A. baumannii* strain 1437282. We studied the phylogenetic distribution of these proteins among closely related genomes (Supplementary Figure S1). Hly III was found in all studied genomes, and all but three genomes have at least one hemolysin homologous proteins coded. HylD is almost completely conserved, only missing in a specific basal lineage. Interestingly, this lineage is characterized not only by the absence of HylD but also for the absence of Hec-B. Hec-B homologous genes seem to be restricted to non-related specific lineages, displaying a non-uniform phylogenetic distribution (Supplementary Figure S1). In addition, other putative hemolytic related proteins were identified in the genome of A47; but annotation data is inconclusive about its role in the hemolytic activity. This is the case of WP_038342744.1, an RND transporter subunit, and WP_038342201.1, a 21 kDa hemolysin precursor with 81% identity to a BON domain-containing protein.

Due to the contribution of T4P to virulence, genes associated with its biogenesis were searched and all T4P associated genes were identified in A47 and are described in Table 1.

**TABLE 1:** Type IV pili genes identified in the A47 genome and predicted function with relation to T4P biogenesis and function.

**Gene**	**Predicted function**
*pilA*	Major pilin
*pilB*	Type 4 fimbrial biogenesis protein
*pilC*	Type 4 fimbrial assembly protein
*pilD*	Type 4 prepilin-like proteins leader peptide processing enzyme
*pilE*	Type 4 pilus assembly protein and pilin like competence factor
*pilF*	Type 4 fimbrial biogenesis protein
*pilG*	Twitching motility two-component system response regulator
*pilH*	Twitching motility two-component system response regulator
*pilI*	Twitching motility protein
*pilJ*	Type 4 pilus biogenesis protein
*pilM*	Type 4 pilus assembly protein
*pilN*	Type 4 pilus assembly protein
*pilO*	Type 4 pilus assembly protein
*pilP*	Type 4 pilus assembly protein
*pilQ*	Type 4 pilus assembly protein
*pilR*	Type 4 fimbriae expression regulatory protein
*pilS*	Sensor protein
*pilT*	Putative type 4 fimbrial biogenesis protein and twitching motility protein
*pilU*	Twitching motility protein
*pilV*	Type 4 fimbrial biogenesis protein
*pilW*	Type 4 pilus assembly protein
*pilX*	Putative type 4 fimbrial biogenesis protein
*pilY1*	Type 4 pilus assembly protein
*pilZ*	Type 4 fimbrial biogenesis protein
*fimB*	Type 4 fimbrial assembly protein
*fimT*	Type 4 pilus assembly protein

*Adapted from a supplemental table originally presented by Antunes et al. (2011).*

**FIGURE 2:**
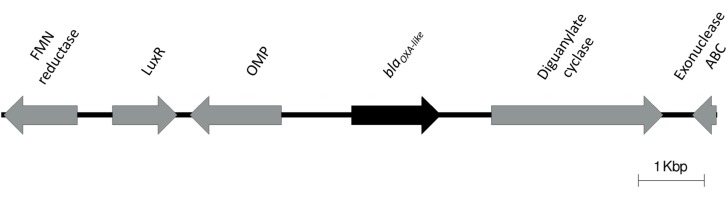
Genetic context of the novel *bla*_OXA–like_ identified in the A47 genome. Direction of the arrows indicate directionality of the coding genes.

A47 is resistant to ampicillin, cefalotin, cefoxitin, and cefotaxime, and exhibits susceptibility to a variety of other antibiotics. Considering that ubiquitous β-lactamases have been reported in all *Acinetobacter* genomes, we searched for β-lactamases in A47 genome. A novel chromosomally located *bla*_OXA–like_ gene (897 bp) was identified (Nucleotide accession number: KT835650). This gene exhibits the highest percent nucleotide identity (79%) to *bla*_OXA–298_ from *Acinetobacter* sp. NIPH 3623 (Accession number: NG_049593). The predicted protein sequence corresponding to this novel β-lactamase is 288 residues and has the highest percent (73%) identity to a class D β-lactamase from *Acinetobacter* sp. YK3 (Accession number: WP_069578983). Additionally, this novel β-lactamase exhibits 72% amino acid identity to a class D β-lactamase from *Acinetobacter* sp. Neg1 (Accession number: WP_047430089.1), OXA-295 from *Acinetobacter* sp. NIPH 2168 (Accession number: WP_005260134.1) and OXA-294 from *Acinetobacter* sp. NIPH 758 (Accession number: WP_004776204.1). The genetic context of the novel β-lactamase is shown in Figure 2. A BLAST search revealed only one isolate of *A. haemolyticus* TJS01 sharing 75% identity to the genetic context (5 kbp upstream and downstream) of the *bla*_OXA–like_ gene.

The molecular evolution of *bla*_OXA–like_ and homologous sequences in closely related genomes was studied. The phylogenetic reconstruction and the estimated amino acid *p*-distance suggest that the *bla*_OXA–like_ protein found in A47 (WP_052209191.1) has significantly diverged from other homologs found in closely related genomes (Supplementary Figure S3). On average the more similar group of *bla*_OXA–like_ proteins has a *p*-distance of 0.245 (G2 in Supplementary Figure S3A). This group is formed for OXA-298 and OXA-297 proteins found in *Acinetobacter* sp. NIPH 3623 and *Acinetobacter* sp. NIPH 1847, respectively. On the other hand, the most distantly related *bla*_OXA–like_ proteins found were OXA-58 proteins. The effect of selection at the molecular level was studied by means of the dN/dS test. Results suggest there is evidence of episodic diversifying selection in the lineage of the *bla*_OXA–like_ gene found in A47. This is also true in three other independent lineages. Significance was assessed using the Likelihood Ratio Test at a threshold of *P* ≤ 0.05, after correcting for multiple testing. The site model dN/dS test found evidence of positive/diversifying selection in two amino acid sites, when considering the whole alignment (Supplementary Figure S3C). Taken together, these results suggest that the *bla*_OXA–like_ protein found in A47 and other homologous proteins found in closely related genomes may be evolving under positive selection. An incomplete aminoglycoside resistance gene (*aac(6′)-Ir*) was also identified. We searched for the presence of efflux pump systems in the A47 genome. AdeIJK system, with 81% identity with *A. junii* strain 65 (AN CP019041) and the AdeABC proteins with 78, 79, and 72% identity, respectively with *A. baumannii* BM4454 (AN AF370885) were found. Two regulatory (*adeS* and *adeR*) genes of the AdeABC system were also found, with 72 and 78% identity with *A. calcoaceticus* NCTC7364 (AN LT605059) and *A. lactucae* OTEC-02 (AN CP020015), respectively. Further genomic analysis revealed the absence of insertion sequences in A47 genome. Using the PHAST tool to predict phage sequences, two intact prophages, one incomplete prophage, and two questionable prophages were identified in A47 genome.

In addition, CRISPR-cas systems were searched and a CRISPR-cas system, which belongs to subtype IF, was identified in A47 genome. The system contains the cluster *cas* genes including the *cas1*, *cas3-cas2*, *csy1*, *csy2*, *csy3*, and *cas* and a 91 spacer (GTTCATGGCGGCATACGCCATTTAGAAA). This variant of Cas-IF type was previously described in other bacterial species such as *Shewanella putrefaciens* and *Yersinia pseudotuberculosis* (Makarova et al., 2018). No toxin-antitoxin systems were identified in A47 genome.

The distribution of the T6SS components in *Acinetobacter* was studied, showing a large variability in the presence or absence of the 13 T6SS core genes in *Acinetobacter* species (Traglia et al., 2018). To address the presence of this system in A47 genome, the thirteen genes coding for the core proteins of this system were searched for within the A47 genome, and their presence was confirmed showing 94–100% amino acid sequence identity against the available genes deposited in the GenBank.

### A47 Can Naturally Acquire Genomic and Plasmid DNA

Considering that the genes required for T4P biogenesis in A47 were found, the implications of T4P in A47 DNA-acquisition were investigated. Transformations, using plasmid and gDNA, were carried out as previously described (Ramirez et al., 2010). A47 was successfully transformed with all DNA sources tested, suggesting that A47 is naturally competent under the tested condition.

Transformation frequencies using two DNA sources (plasmid and gDNA) were calculated in A47. We observed that A47 can be transformed with DNA both sources. Transforming with gDNA resulted in A47::A33405 and A47::A144 with transformation frequencies of 1.19 × 10^–6^ TF/CFU (SD ± 7.83 × 10^–7^) and 3.07 × 10^–6^ TF/CFU (SD ± 3.14 × 10^–6^), respectively. Plasmid transformation frequencies were 7.20 × 10^–7^ TF/CFU (SD ± 1.96 × 10^–7^) and 3.56 × 10^–6^ TF/CFU (SD ± 3.61 × 10^–6^) for A47::pDSRed and A47::pJHCMW1, respectively.

Randomly selected transformant colonies were picked and assessed for changes in resistance profile using a disk diffusion screening method. We observed a variety of changes in the susceptibility profile of the transformant colonies (data not shown). To further determine the level of susceptibility of A47 and transformants, MIC was performed. The most substantial increase in MIC was observed with AK and GN for A47::Ab33405 (Table 2).

**TABLE 2:** Comparison of A47 and transformed A47 MIC’s.

**Strain**	**AK**	**CAZ**	**GN**	**CT**
A47	1.5	1.0–1.5	0.19–0.25	1.5
A47::A33405	**16–24**	1.5–2.0	3.0–4.0	6.0
A47::A144	n/a	1.5	1.0–1.5	n/a
A47::pDSRed	6.0	1.0–1.5	**6.0**	n/a

*Following the 2017 CLSI breakpoints for *Acinetobacter* spp. MIC’s are expressed as μg/mL. Bold numbers represent intermediate susceptibility levels.*

### A47 Can Form Biofilms on Two Different Abiotic Surfaces and Exhibits Type IV Pili (T4P) Like Structures on Its Cell Surface

**FIGURE 3:**
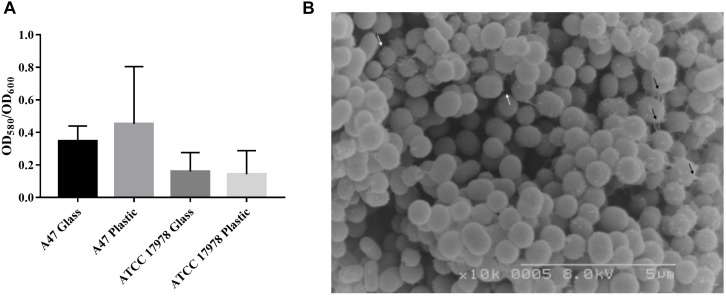
**(A)** Amount of biofilm formed by A47 and ATCC 17978 on polystyrene plastic or borosilicate glass reported as a ratio of biofilm (OD580) to total biomass (OD600). Difference in amount of biofilm formed relative to biomass are not statistically significant (*P* > 0.05) between the two abiotic surfaces as evaluated using a paired Student’s *t*-test. Means for each condition are represented by black bars (*n* = 3). **(B)** SEM magnified (10×) image of A47 exhibits a coccobacilli shape and surface structures which vary in length. Longer cellular appendages are indicated by black arrows and white arrows indicate short pili-like appendages.

A47 ability to form biofilms on both polystyrene plastic and borosilicate glass abiotic surfaces was observed (Figure 3A). As *A. baumannii* ATCC 17978 is a known biofilm producer, this strain was used as an experimental control as well as a means of comparison for A47 biofilm production (Nait Chabane et al., 2014). A47 forms biofilms on both polystyrene plastic and borosilicate glass abiotic surfaces (Figure 3A). On average, A47 produces a larger amount of biofilm on polystyrene plastic than borosilicate glass (Figure 3A). Comparison of A47 OD_580_/OD_600_ on the two abiotic surfaces is not statistically significant (*P* > 0.05) by a two-tailed Mann–Whitney *U* test. Comparison between A47 and ATCC 17978 OD_580_/OD_600_ did not yield statistically significant results (*P* > 0.05) as determined by an ANOVA test under the tested conditions (Figure 3B).

Upon identification of all T4P genes in A47, SEM was used to visualize the surface of A47 cells. SEM images of A47 exhibit multiple surface appendages and a coccobacilli shape. The appendages vary in size and distribution; however, the most common and distinct appendages are long cellular extensions and shorter potential pili-like structures unevenly distributed (Figure 3B). Longer appendages are variable in length and appear to connect individual bacteria or anchor bacteria (Figure 3B). Short pili-like appendages are The coccobacilli shape is in line with what has previously been observed for *Acinetobacter*.

### A47 Exhibits a High Hemolytic Activity

**FIGURE 4:**
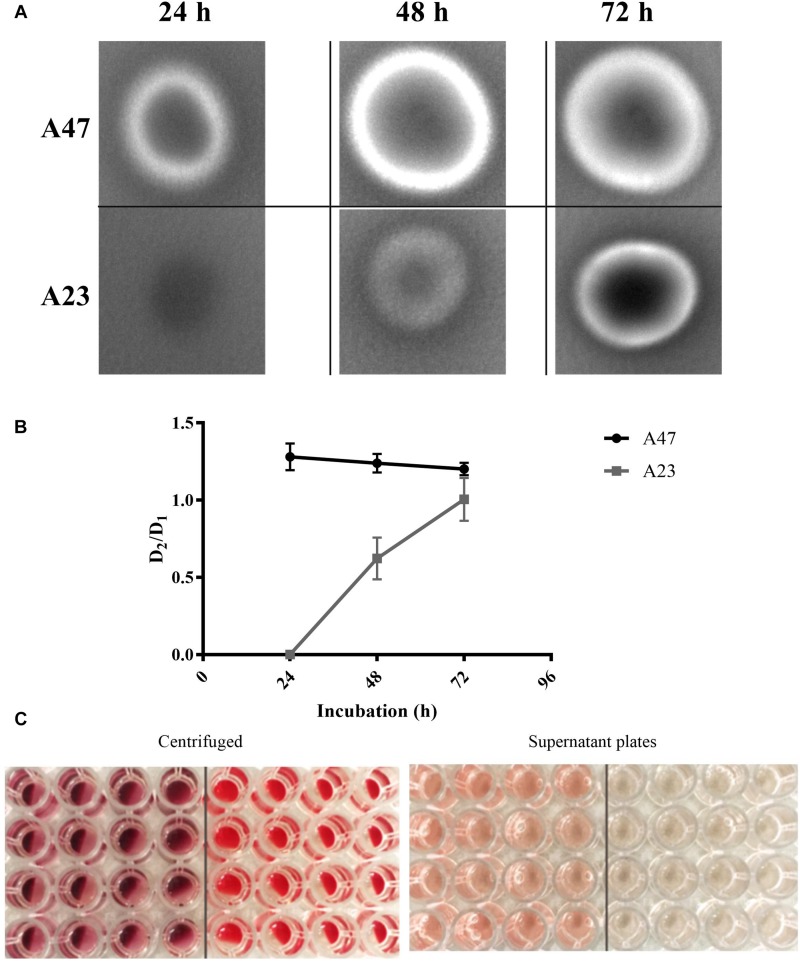
**(A)** Observed hemolytic activity in A47 and A23. TSA supplemented with 5% rabbits’s blood was inoculated with A47 and *A. haemolyticus* A23, incubated at 37°C and imaged at 24, 48, and 72 h post-inoculation. A47 and A23 exhibit β-hemolysis generating a zone of clearing around colonies, representative of complete lysis of red blood cells. **(B)** Quantification A47 and A23 hemolysis. Blood agar plates were aseptically inoculated with colonies of A47 and A23, and incubated at 37°C. The diameters of lytic zones (D2) and colonies (D1) was measured at 24, 48, and 72 h. Hemolysis is reported as the average ratio (D2/D1), data points represent individual ratios for each biological samples (*n* = 16). The curve intersects at the average D2/D1 for each time point. **(C)** A47 exhibits visible signs of hemolytic activity. Compared to LB conditions on right half, A47 wells exhibit visible signs of hemolytic activity, evidenced in both centrifuged (C1) and supernatant plates (C2). The deep red color change indicates hemolytic activity in (C1), and the effect of lysed blood cells bleeding out into the supernatant is shown in (C2).

With the aim to characterize A47 hemolytic activity, two different approaches were performed. As *A. haemolyticus* is a well-studied hemolytic species of *Acinetobacter*, the *A. haemolyticus* strain A23 was used as a positive control for β-hemolysis. Only A47 showed lysis of red blood cells after 24 h, as indicated by a zone of clearing surrounding the colony. A23 did not exhibit β-hemolysis until 48 h post-inoculation (Figure 4A). When using defibrinated rabbit blood in a 96-well microplate, A47 was shown to exhibit hemolytic activity. After 12 h, there was significant hemolytic activity in A47 conditions, in contrast to the control negative LB conditions (*P* < 0.005) (Figure 4B). Hemolytic activity of A47 bacteria was also visible based on the color change of the wells (Figure 4C).

### A47 Exhibits Photoregulation of Different Virulence Traits

**FIGURE 5:**
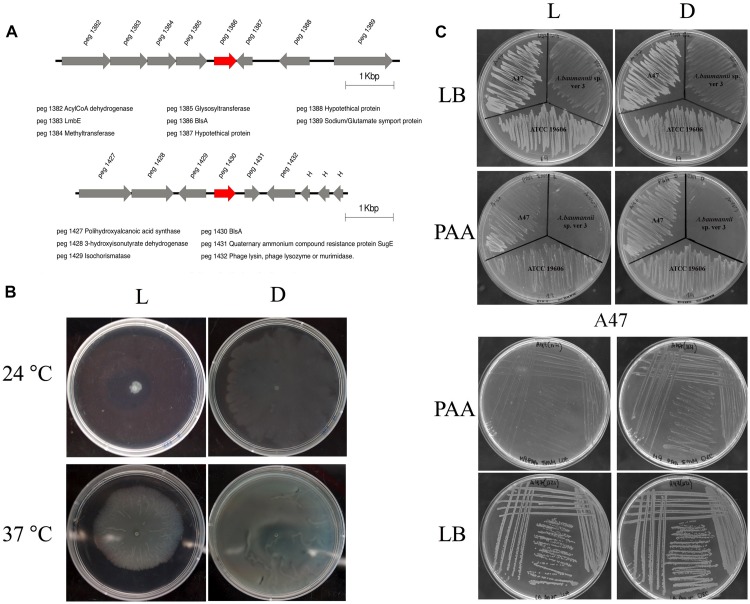
**(A)** Genomic context of BLUF-domain containing genes in *Acinetobacter* sp. A47. Gene annotations are indicated below the scheme. In red are indicated BLUF photoreceptors. The figure was constructed using information retrieved from RAST and the seed viewer. **(B)** Effects of light and temperature on motility. Cells of *Acinetobacter* sp. A47 were inoculated on the surface of motility plates. Plates were inspected and photographed after overnight incubation in darkness (D) or in the presence of blue light (L) at 24°C or 37°C. **(C)** Light inhibits growth of *Acinetobacter* sp. A47 in PAA. Growth of *Acinetobacter* sp. A47 in M9 minimal agar medium supplemented with 5 mM PAA as a sole carbon source and incubated stagnantly at 24°C under blue light or in the dark. Also shown is growth of *A. baumannii* ATCC 19606 and *Acinetobacter* sp. ver 3 as controls, as well as growth in LB to show no effect of light on viability of the strains. Shown are representative results obtained from three independent experiments.

Analysis of the A47 genome reveals the presence of two BLUF-type photoreceptors (Figure 5A), one of which – peg 1386 – is 82% identical with respect to the BlsA homolog present in *A. baumannii* ATCC 17978 strain. This putative photoreceptor is globally synthenic to the genes located upstream with respect to *blsA*, showing differences mostly in the length of the intergenic region. On the contrary, no conservation is observed in the genes located downstream to *blsA*, except peg 1389 which codes for a putative sodium/glutamate symport protein. The second BLUF-coding protein, peg 1430, shows 52% identity respect to *A. baumannii* ATCC 17978 BlsA. It is flanked by a putative isochorismatase (peg 1429), enzymes involved in the synthesis of 2,3-dihydroxy-2,3-dihydrobenzoate and pyruvate from isochorismate, which are frequently involved in siderophore synthesis. Located next to this gene is a putative 3-hydroxyisobutyrate dehydrogenase (peg 1428), which participates in leucine, valine and isoleucine degradation, and there is a gene coding for a putative polihydroxyalcanoic acid synthase (peg 1427). 3′ downstream from the *blsA* homolog, there is a gene coding for a quaternary ammonium compound resistance protein (peg 1431), followed by a phage lysin coding gene (peg 1432).

We have studied whether A47 can sense and respond to light by studying different cellular processes. As shown in Figure 5B, A47 exhibits photoregulation of motility at 24°C. Motility was inhibited under blue light, while the bacteria grew and spread over the surface of the plate in the dark. At 37°C, A47 still shows photoregulation of motility, although to a lesser extent than at 24°C. In addition, we studied other traits previously shown to be photoregulated in *A. baumannii*, such as metabolism (Muller et al., 2017). Figure 5C shows that light modulates the PAA utilization pathway at 24°C, just as it does in *A. baumannii* (Muller et al., 2017). On the contrary, we were not able to detect photoregulation of trehalose biosynthesis (data not shown).

## Discussion

The genus *Acinetobacter* represents an important group of pathogens mainly due to the extreme genome plasticity and the ability to acquire foreign DNA.

Our results, including phylogenetic and comparative genomic analysis, indicate that *Acinetobacter* A47 represents a new species. More importantly, according to the phylogenetic and the ANI analyses, we found that many strains with closely related genomes are likely misidentified: *A. gyllenbergii*, *A. colistiniresistens*, *A. proteolyticus*, *A. parvus*, *A. tjernbergiae*, and *A. beijerinckii*.

When analyzing genes associated with pathogenesis, four genes putatively involved in hemolytic activity were identified in the genome of A47. There is significant documented variation in hemolysis in the *Acinetobacter* genus, with the most uncommon type of hemolytic activity being the β-hemolysis (Tayabali et al., 2012; Dahdouh et al., 2016). Our results show that the novel species A47 exhibits β-hemolysis, falling within a small subset of β-hemolytic *Acinetobacter* species. Analyses of hemolysis-related genes and their distribution showed that three of the putative genes are highly conserved in the studied genomes (Supplementary Figure S1). However, Hec-B-like gene was identified in different and independent lineages, displaying a patchy phylogenetic distribution. Thus, many of the analyzed genomes, which share the four analyzed hemolysis-related homologs, may also exhibit β-hemolysis activity, though none have currently been reported in the literature.

In addition, genomic analysis revealed the presence of a novel OXA-like β-lactamase gene. This OXA-like gene is unique and may be ubiquitous in this novel species, but more isolates will need to be recovered before this can be determined. No signs of pseudogenization has been found in this gene, and at least one fourth of the amino acids residues in this protein are different to its closest related homologs found in the studied genomes. Note, however, that many putative functional residues are also conserved in this protein (Supplementary Figure S3C). The observed highly divergence may in part be explained in the observed susceptibility to the tested β-lactams. Interestingly, molecular evolution tests found evidence of positive selection driving the evolution of the coding sequence of this new *bla*_OXA–like_ gene. This suggests that amino acid changes may be related to a functional change of the coded protein. Another possible scenario is that this gene is not derived from OXA-like homologs found in closely related genomes. In other words, the observed divergence could be explained by a horizontal transfer event. This scenario, although not entirely improbable, is not compatible with the observation that highly similar sequences in public databases belong to closely related genomes (data not shown). Future research into this novel β-lactamase could investigate the affinity of this enzyme to different β-lactams and/or possible novel functions. In addition, we performed molecular docking studies to assess the mechanism of A47 OXA-like and we observed that this enzyme has similar binding affinity against imipenem and doripenem comparing with OXA-51, and stronger affinity for oxacillin (Supplementary Figure S4 and Supplementary Table S4). Further phenotypic studies are needed to confirm this observation.

Genes coding for T4P were found in A47’ genome and its ability to naturally acquire different types of DNA was also confirmed. Acquisition of exogenous DNA is not specific to the nature of DNA, as evidenced by the bacteria’s ability to acquire plasmid and gDNA fragments. This was previously observed in *A. calcoaceticus* BD413 (also referred to as *A. baylyi* ADP1), and the same system of DNA uptake was found to be utilized in both instances (Palmen et al., 1993). The mechanism A47 uses to uptake foreign DNA was not examined in depth, although T4P have previously been implicated in the acquisition of exogenous DNA (Harding et al., 2013). It is interesting to note that there is no significant difference between transformation frequencies when A47 was transformed with plasmid or gDNA. Furthermore, biofilm formation was also observed in A47 on plastic and glass. Comparisons of A47 biofilm formation on polystyrene plastic or borosilicate glass was not statistically significant (*P* > 0.05). Additionally, no statistically significant differences between A47 biofilm formation and ATCC 17978 biofilm formation were observed between the tested surfaces (*P* > 0.05). ATCC 17978 has been characterized as a weak biofilm producer, and the findings here suggest that A47 is a poor biofilm producer as well. Images obtained using SEM showed the presence of multiple cellular surface appendages of variable length. De Breij and colleagues similarly examined surface structures of *A. baumannii* and identified long cellular-like projections as well as potential pili-like surface structures (De Breij et al., 2010). Although these results do not confirm that these surface structures are T4P, they provide substantial evidence that A47 does express non-uniform surface appendages. These images combined with the genetic identification of all the T4P genes within the A47 genome support the hypothesis that A47 expresses T4P.

The T6SS was recently recognized as a key system for bacterial competition and involved in HGT (Cooper et al., 2017; Ringel et al., 2017). Genomic results showed the presence of the T6SS genomic locus in A47, which could contribute to its repertoire of potential virulent traits.

Additionally, *A. baumannii* and other members of the *Acinetobacter* genus have been shown to sense and respond to light (Mussi et al., 2010; Muller et al., 2017). While various BLUF type photoreceptors are present in non*-baumannii* species, BlsA is the only BLUF protein that has been detected in *A. baumannii*. Our data indicates the presence of two BLUF-type photoreceptors in the genome of *Acinetobacter* sp. A47, and we have verified the ability of this strain to photoregulate both motility as well as the utilization of PAA.

## Conclusion

The work presented here provides an initial characterization of some of A47’s virulence factors and demonstrates why A47 represents an important new species of *Acinetobacter*. The general phylogenetic background and specific aspects of this isolate’s evolution were studied. The genome of A47 harbors a novel β-lactamase is susceptible to all tested antibiotics, forms biofilms, can naturally acquire foreign DNA, is a member of a small group of *Acinetobacter* that carry out β-hemolysis and possesses the genes coding for the T6SS, and has the ability to sense and response to light, regulating motility and PAA catabolism. This novel species of *Acinetobacter* can greatly improve the current understanding of this genus due to its unique characteristics.

## Data Availability

All datasets generated for this study are included in the manuscript and/or the Supplementary Files.

## Author Contributions

STJS, AI, and MSR conceived the study and designed the experiments. STJS, KP, SM, SF, JSF, MRT, GMT, MAM, AI, MA2, and MSR performed the experiments and genomics and bioinformatics analyses. STJS, SM, SF, JSF, GMT, MAM, MA1, MA2, MRT, AI, and MSR analyzed the data and interpreted the results. MAM, AI, and MSR contributed reagents, materials, and analysis tools. MA1 and CV contributed with the strains. STJS, SF, SM, MRT, MA, AI, and MSR wrote the manuscript. All authors read and approved the final manuscript.

## Conflict of Interest Statement

The authors declare that the research was conducted in the absence of any commercial or financial relationships that could be construed as a potential conflict of interest.

## Footnotes

1 http://www.bacterio.net/acinetobacter.html

2 www.ncbi.nlm.nih.gov/BLAST

## Supplementary Material

FIGURE S1 Phylogenetic distribution of putative hemolysis-related genes found in closely related genomes studied. A47 genome is indicated with a black arrow. The visualization was generated in the iTOL web server available at itol.embl.de.

FIGURE S2 Approximate maximum likelihood phylogenetic tree of A47 and closely related assemblies. The phylogenetic tree was build based on 1383 concatenated orthologous proteins. The tree was inferred using FastTree version 2.1, with LG + G model. The SH-like test was used to evaluate branch supports and indicated as red values next to nodes. Genomes from the same species based on two-way ANI score (>95%) were indicated with brackets. The position of A47 is indicated with a black arrow.

FIGURE S3 Molecular evolution and sequence analyses of *bla*_OXA–like_ homologs. **(A)** Maximum likelihood phylogenetic reconstruction of homologs found in closely related genomes. The phylogenetic tree was build based on proteins sequences using the PHYML version 3.1 software, with the LG + G model. The SH-like test was used to evaluate branch supports. Groups are indicated next to basal nodes with “G.” The position of *bla*_OXA–like_ gene found in A47 is indicated with a black arrow. Evidence of positive selection was found in four lineages, indicated in magenta. **(B)** Average amino acid *p*-distance estimated between monophyletic groups in the lower triangular matrix, standard deviation of the average estimation in the upper triangular matrix. Groups are defined in phylogeny shown in panel **(A)**. **(C)** Clustal-omega alignment of OXA-like proteins ordered according to monophyletic groups, as stated in A. Two sites evolving under positive selection are indicated with black arrows.

FIGURE S4 Computed two-dimensional structure of the OXA-like (UCSF Chimera 1.12) and the interaction between OXA-like and oxacillin. **(A)** Ribbon structure of OXA-like colored in rainbow style. **(B)** Surface topology of OXA-like colored by Coulombic surface coloring (UCSF Chimera 1.12). Surface was calculated using AMBER ff 14SB charge model on three color scale of red, −10 kcal/(mol e), white, 0 kcal/(mol e), and blue, 10 kcal/(mol e), where e is unit electron charge. **(E)** Ramachandran plot analysis of the OXA-like structure. Binding of various antibiotic molecules with **(C)** the OXA-like and **(D)** OXA-51. Different antibiotics was colored in violet and residues was colored in orange. H-bond lines between different residues and the ligands was colored in green.

TABLE S1 List of 56 genomes used in the present study.

TABLE S2 List of putative unique genes in *Acinetobacter* sp. 47.

TABLE S3 Two-way average nucleotide identity estimated among closely related genomes that belongs to the A47 strain monophylogenetic cluster.

TABLE S4 Predicted binding affinity between OXA-like from *Acinetobacter* sp. A47 and OXA-51 from *A. baumannii* against to doripenem, imipenem, and oxacillin.
