# Supplementary figures and images for: Genetic and Phenotypic Features of a Novel Acinetobacter Species, Strain A47, Isolated From the Clinical Setting
Source: Front Microbiol. 2019 Jun 18;10:1375. doi: 10.3389/fmicb.2019.01375 (PMC6591377; doi:10.3389/fmicb.2019.01375)

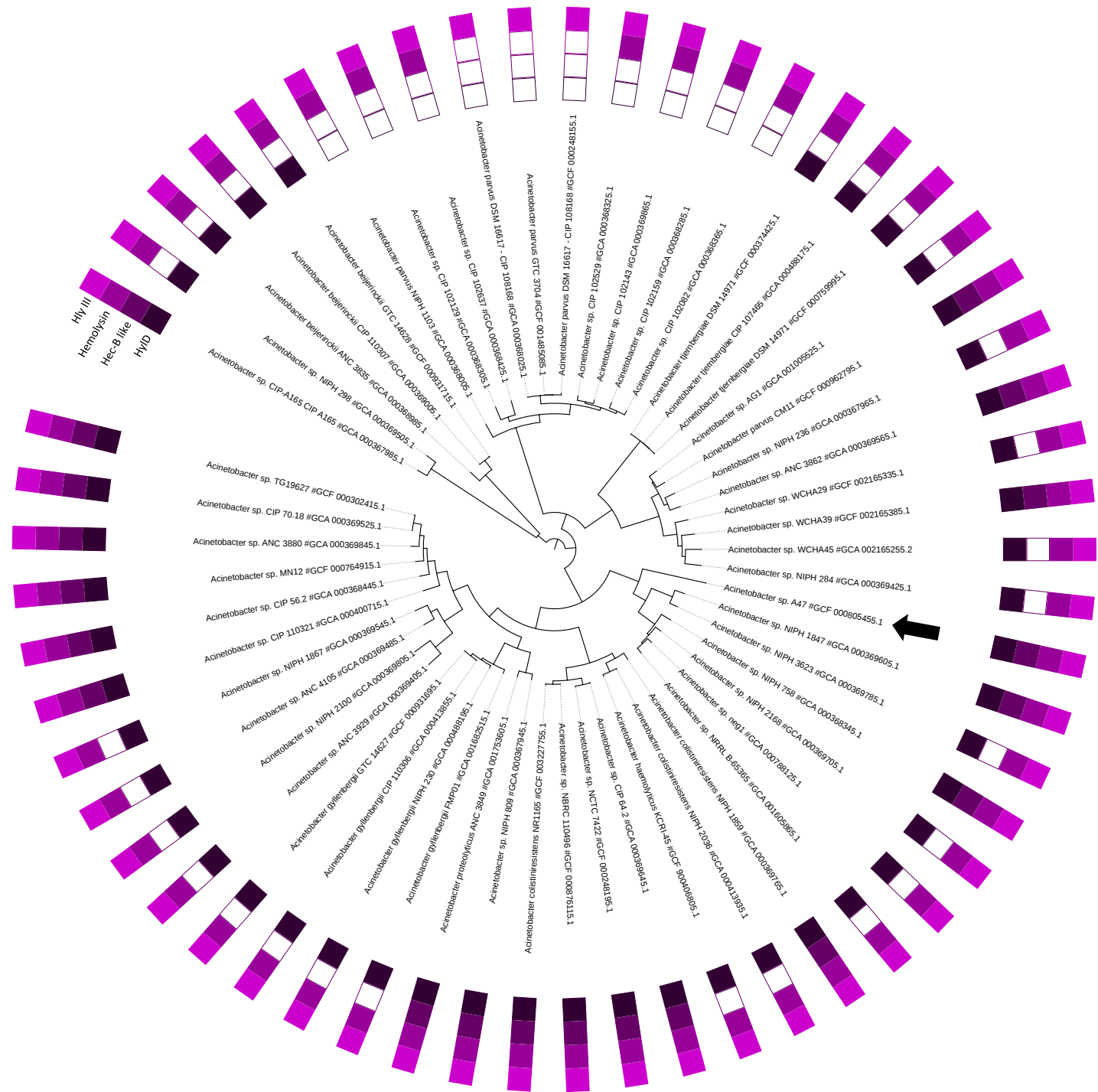

Supplement: FIGURE S1 — Phylogenetic distribution of putative hemolysis-related genes found in closely related genomes studied. A47 genome is indicated with a black arrow. The visualization was generated in the iTOL web server available at itol.embl.de. [file Image_1.pdf]

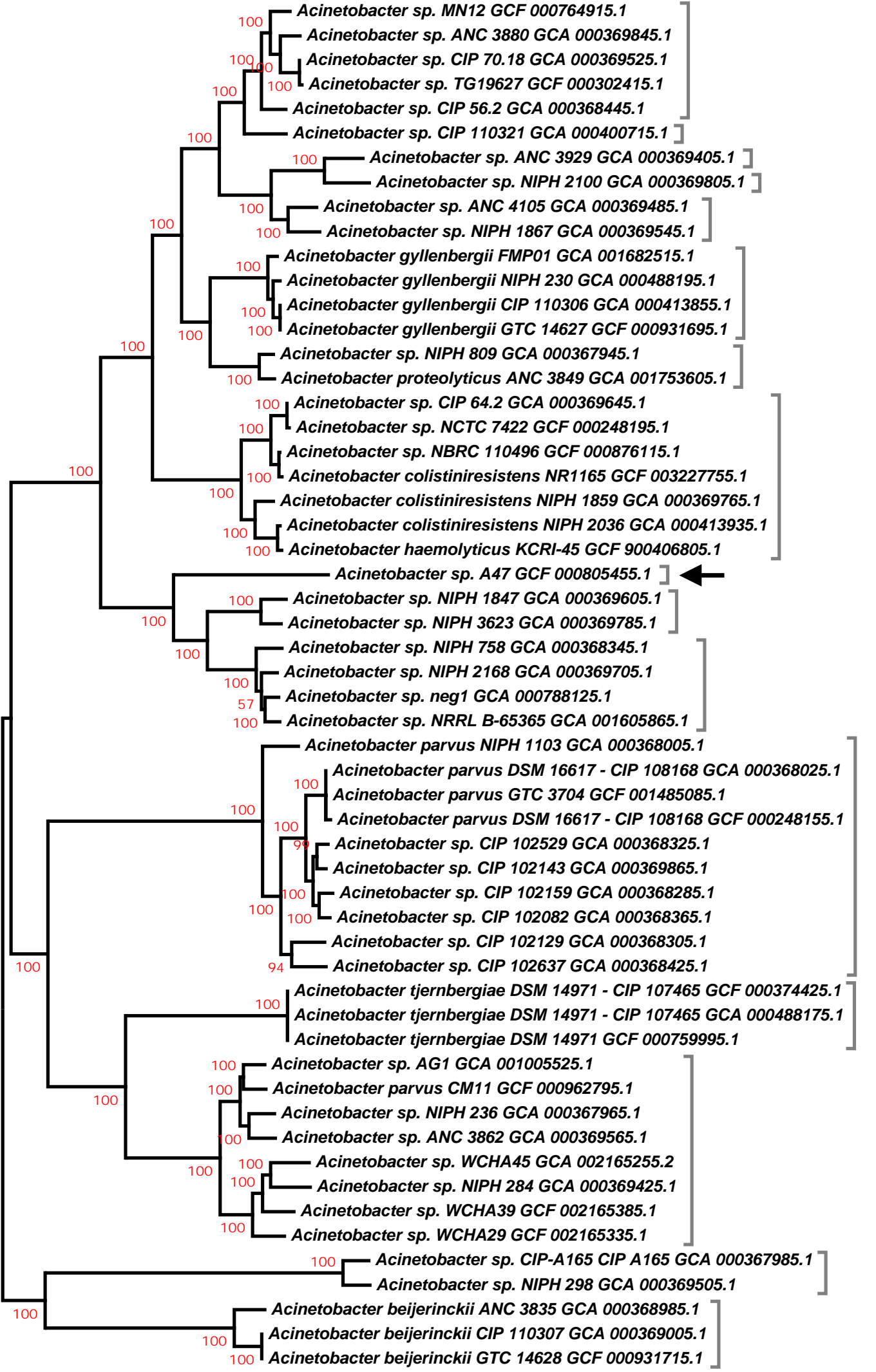

0.01

Supplement: FIGURE S2 — Approximate maximum likelihood phylogenetic tree of A47 and closely related assemblies. The phylogenetic tree was build based on 1383 concatenated orthologous proteins. The tree was inferred using FastTree version 2.1, with LG + G model. The SH-like test was used to evaluate branch supports and indicated as red values next to nodes. Genomes from the same species based on two-way ANI score (>95%) were indicated with brackets. The position of A47 is indicated with a black arrow. [file Image_2.pdf]
