# Supplementary material for: Genetic and Phenotypic Features of a Novel Acinetobacter Species, Strain A47, Isolated From the Clinical Setting
Source: Front Microbiol. 2019 Jun 18;10:1375. doi: 10.3389/fmicb.2019.01375 (PMC6591377; doi:10.3389/fmicb.2019.01375)
Supplement: FIGURE S3 — Molecular evolution and sequence analyses of blaOXA–like homologs. (A) Maximum likelihood phylogenetic reconstruction of homologs found in closely related genomes. The phylogenetic tree was build based on proteins sequences using the PHYML version 3.1 software, with the LG + G model. The SH-like test was used to evaluate branch supports. Groups are indicated next to basal nodes with “G.” The position of blaOXA–like gene found in A47 is indicated with a black arrow. Evidence of positive selection was found in four lineages, indicated in magenta. (B) Average amino acid p-distance estimated between monophyletic groups in the lower triangular matrix, standard deviation of the average estimation in the upper triangular matrix. Groups are defined in phylogeny shown in panel (A). (C) Clustal-omega alignment of OXA-like proteins ordered according to monophyletic groups, as stated in A. Two sites evolving under positive selection are indicated with black arrows. [file Image_3.pdf]

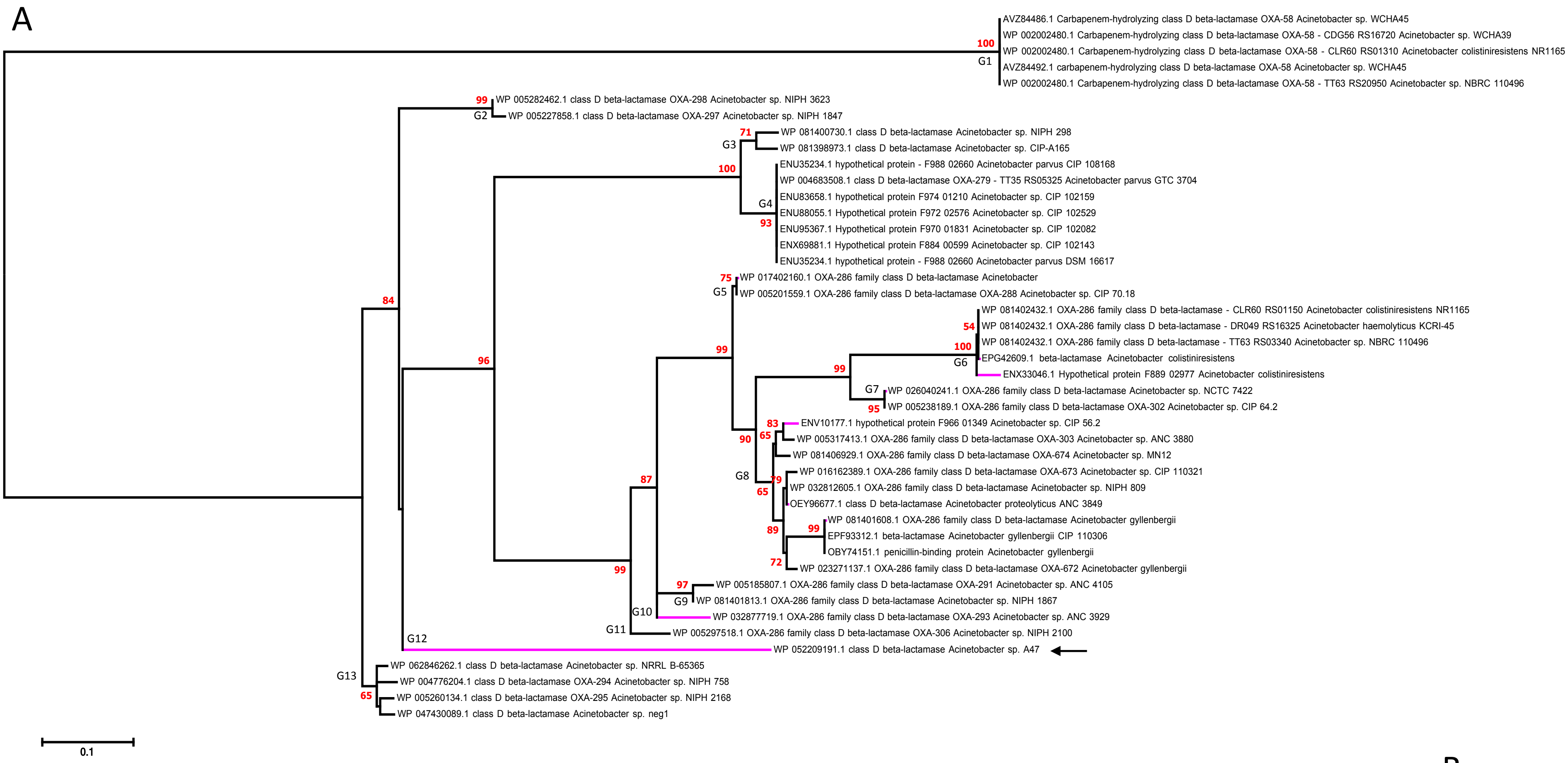

B

**G1**		*0.029*	*0.029*	*0.028*	*0.029*	*0.029*	*0.029*	*0.029*	*0.029*	*0.029*	*0.029*	*0.029*	*0.029*
**G13**	0.467		*0.019*	*0.026*	*0.026*	*0.027*	*0.024*	*0.026*	*0.026*	*0.024*	*0.024*	*0.025*	*0.023*
**G2**	0.471	0.117		*0.026*	*0.027*	*0.027*	*0.026*	*0.027*	*0.027*	*0.025*	*0.025*	*0.026*	*0.025*
**G12**	0.475	0.256	0.245		*0.030*	*0.030*	*0.027*	*0.028*	*0.027*	*0.028*	*0.027*	*0.028*	*0.027*
**G3**	0.490	0.261	0.255	0.356		*0.014*	*0.026*	*0.025*	*0.026*	*0.025*	*0.025*	*0.026*	*0.025*
**G4**	0.486	0.263	0.253	0.346	0.070		*0.027*	*0.026*	*0.027*	*0.025*	*0.027*	*0.027*	*0.026*
**G5**	0.486	0.222	0.224	0.307	0.272	0.276		*0.021*	*0.018*	*0.013*	*0.014*	*0.018*	*0.017*
**G6**	0.482	0.251	0.254	0.322	0.280	0.272	0.144		*0.018*	*0.019*	*0.021*	*0.023*	*0.023*
**G7**	0.479	0.252	0.249	0.319	0.298	0.292	0.086	0.113		*0.016*	*0.020*	*0.022*	*0.022*
**G8**	0.480	0.230	0.229	0.319	0.272	0.267	0.069	0.135	0.091		*0.017*	*0.020*	*0.019*
**G9**	0.494	0.221	0.220	0.305	0.272	0.278	0.062	0.146	0.128	0.108		*0.016*	*0.016*
**G10**	0.494	0.235	0.239	0.331	0.284	0.288	0.086	0.167	0.144	0.130	0.076		*0.018*
**G11**	0.490	0.197	0.216	0.292	0.253	0.257	0.089	0.148	0.148	0.132	0.078	0.097	

C

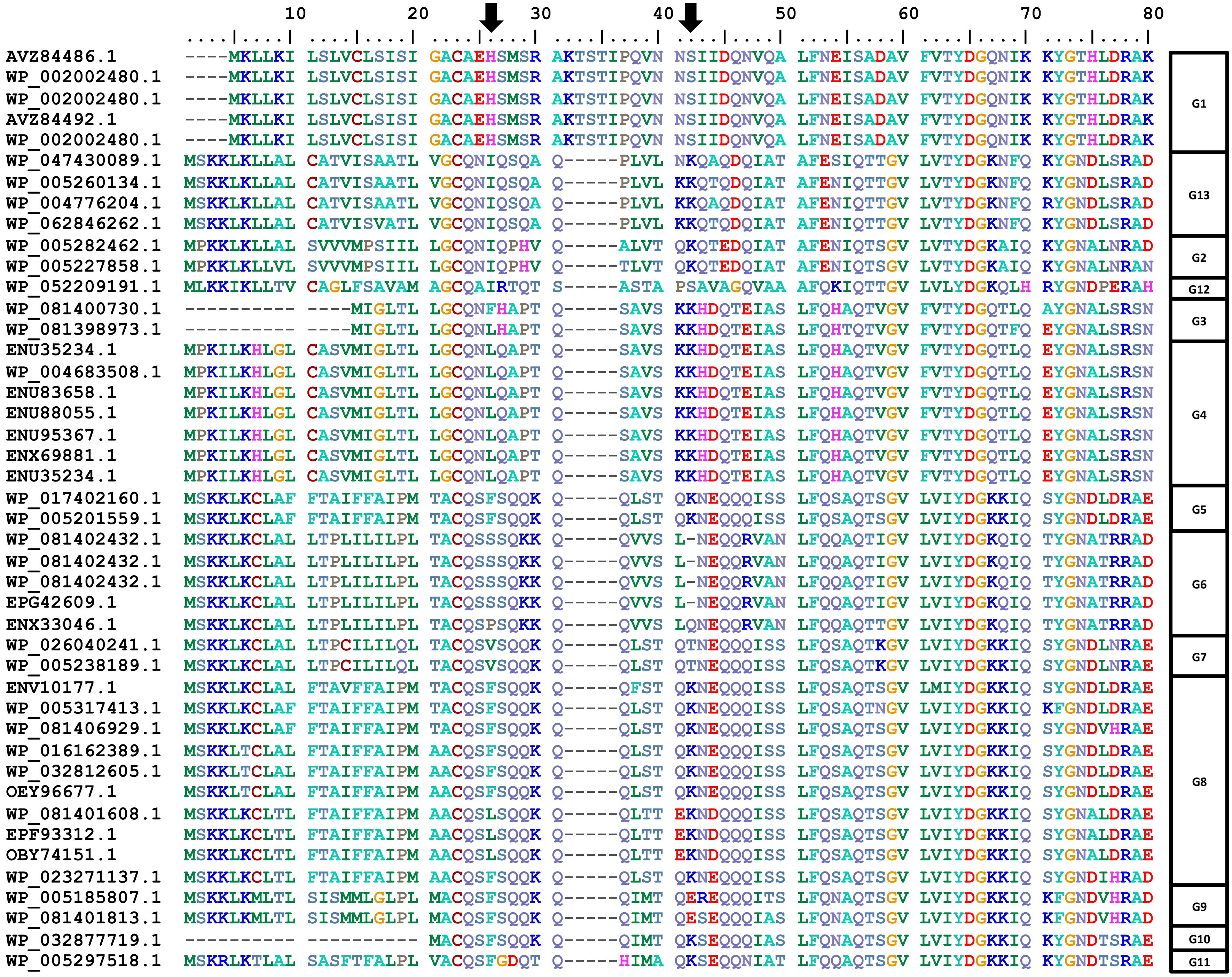
10 20 30 40 50 60 70 80
AVZ84486.1 ----MKLLKI LSLVCLSISI GACAEHSMSR AKTSTIPQVN NSIIDQNVQA LFNEISADAV FVTYDGQNIK KYGTHLDRAK
WP_002002480.1 ----MKLLKI LSLVCLSISI GACAEHSMSR AKTSTIPQVN NSIIDQNVQA LFNEISADAV FVTYDGQNIK KYGTHLDRAK
WP_002002480.1 ----MKLLKI LSLVCLSISI GACAEHSMSR AKTSTIPQVN NSIIDQNVQA LFNEISADAV FVTYDGQNIK KYGTHLDRAK
AVZ84492.1 ----MKLLKI LSLVCLSISI GACAEHSMSR AKTSTIPQVN NSIIDQNVQA LFNEISADAV FVTYDGQNIK KYGTHLDRAK
WP_002002480.1 ----MKLLKI LSLVCLSISI GACAEHSMSR AKTSTIPQVN NSIIDQNVQA LFNEISADAV FVTYDGQNIK KYGTHLDRAK
WP_047430089.1 MSKKLKLLAL CATVISAATL VGCQNIQSQA Q-----PLVL NKQAQDQIAT AFESIQTTGV LVTYDGKNFQ KYGNDLSRAD
WP_005260134.1 MSKKLKLLAL CATVISAATL VGCQNIQSQA Q-----PLVL KKQTQDQIAT AFENIQTTGV LVTYDGKNFQ KYGNDLSRAD
WP_004776204.1 MSKKLKLLAL CATVISAATL VGCQNIQSQA Q-----PLVL KKQAQDQIAT AFENIQTTGV LVTYDGKNFQ RYGNDLSRAD
WP_062846262.1 MSKKLKLLAL CATVISVATL VGCQNIQSQA Q-----PLVL KKQTQDQIAT AFENIQTTGV LVTYDGKNFQ KYGNDLSRAD
WP_005282462.1 MPKKLKLLAL SVVVMPSIIL LGCQNIQPHV Q-----ALVT QKQTEDQIAT AFENIQTSGV LVTYDGKAIQ KYGNALNRAD
WP_005227858.1 MPKKLKLLVL SVVVMPSIIL LGCQNIQPHV Q-----TLVT QKQTEDQIAT AFENIQTSGV LVTYDGKAIQ KYGNALNRAN
WP_052209191.1 MLKKIKLLTV CAGLFSAVAM AGCQAIRTQT S-----ASTA PSAVAGQVAA AFQKIQTTGV LVLYDGKQLH RYGNDPERAH
WP_081400730.1 ---------- ----MIGLTL LGCQNFHAPT Q-----SAVS KKHDQTEIAS LFQHAQTVGV FVTYDGQTLQ AYGNALSRSN
WP_081398973.1 ---------- ----MIGLTL LGCQNLHAPT Q-----SAVS KKHDQTEIAS LFQHTQTVGV FVTYDGQTFQ EYGNALSRSN
ENU35234.1 MPKILKHLGL CASVMIGLTL LGCQNLQAPT Q-----SAVS KKHDQTEIAS LFQHAQTVGV FVTYDGQTLQ EYGNALSRSN
WP_004683508.1 MPKILKHLGL CASVMIGLTL LGCQNLQAPT Q-----SAVS KKHDQTEIAS LFQHAQTVGV FVTYDGQTLQ EYGNALSRSN
ENU83658.1 MPKILKHLGL CASVMIGLTL LGCQNLQAPT Q-----SAVS KKHDQTEIAS LFQHAQTVGV FVTYDGQTLQ EYGNALSRSN
ENU88055.1 MPKILKHLGL CASVMIGLTL LGCQNLQAPT Q-----SAVS KKHDQTEIAS LFQHAQTVGV FVTYDGQTLQ EYGNALSRSN
ENU95367.1 MPKILKHLGL CASVMIGLTL LGCQNLQAPT Q-----SAVS KKHDQTEIAS LFQHAQTVGV FVTYDGQTLQ EYGNALSRSN
ENX69881.1 MPKILKHLGL CASVMIGLTL LGCQNLQAPT Q-----SAVS KKHDQTEIAS LFQHAQTVGV FVTYDGQTLQ EYGNALSRSN
ENU35234.1 MPKILKHLGL CASVMIGLTL LGCQNLQAPT Q-----SAVS KKHDQTEIAS LFQHAQTVGV FVTYDGQTLQ EYGNALSRSN
WP_017402160.1 MSKKLKCLAF FTAIFFAIPM TACQSFSQQK Q-----QLST QKNEQQQISS LFQSAQTSGV LVIYDGKKIQ SYGNDLDRAE
WP_005201559.1 MSKKLKCLAF FTAIFFAIPM TACQSFSQQK Q-----QLST QKNEQQQISS LFQSAQTSGV LVIYDGKKIQ SYGNDLDRAE
WP_081402432.1 MSKKLKCLAL LTPLILILPL TACQSSSQKK Q-----QVVS L-NEQQRVAN LFQQAQTIGV LVIYDGKQIQ TYGNATRRAD
WP_081402432.1 MSKKLKCLAL LTPLILILPL TACQSSSQKK Q-----QVVS L-NEQQRVAN LFQQAQTIGV LVIYDGKQIQ TYGNATRRAD
WP_081402432.1 MSKKLKCLAL LTPLILILPL TACQSSSQKK Q-----QVVS L-NEQQRVAN LFQQAQTIGV LVIYDGKQIQ TYGNATRRAD
EPG42609.1 MSKKLKCLAL LTPLILILPL TACQSSSQKK Q-----QVVS L-NEQQRVAN LFQQAQTIGV LVIYDGKQIQ TYGNATRRAD
ENX33046.1 MSKKLKCLAL LTPLILILPL TACQSPSQKK Q-----QVVS LQNEQQRVAN LFQQAQTTGV LVIYDGKQIQ TYGNATRRAD
WP_026040241.1 MSKKLKCLAL LTPCILILQL TACQSVSQQK Q-----QLST QTNEQQQISS LFQSAQTKGV LVIYDGKKIQ SYGNDLNRAE
WP_005238189.1 MSKKLKCLAL LTPCILILQL TACQSVSQQK Q-----QLST QTNEQQQISS LFQSAQTKGV LVIYDGKKIQ SYGNDLNRAE
ENV10177.1 MSKKLKCLAL FTAVFFAIPM TACQSFSQQK Q-----QFST QKNEQQQISS LFQSAQTSGV LMIYDGKKIQ SYGNDLDRAE
WP_005317413.1 MSKKLKCLAF FTAIFFAIPM TACQSFSQQK Q-----QLST QKNEQQQISS LFQSAQTNGV LVIYDGKKIQ KFGNDLDRAE
WP_081406929.1 MSKKLKCLAF FTAIFFAIPM TACQSFSQQK Q-----QLST QKNEQQQISS LFQSAQTSGV LVIYDGKKIQ SYGNDVHRAE
WP_016162389.1 MSKKLTCLAL FTAIFFAIPM AACQSFSQQK Q-----QLST QKNEQQQISS LFQSAQTSGV LVIYDGKKIQ SYGNDLDRAE
WP_032812605.1 MSKKLTCLAL FTAIFFAIPM AACQSFSQQK Q-----QLST QKNEQQQISS LFQSAQTSGV LVIYDGKKIQ SYGNDLDRAE
OEY96677.1 MSKKLTCLAL FTAIFFAIPM AACQSFSQQK Q-----QLST QKNEQQQISS LFQSAQTSGV LVIYDGKKIQ SYGNDLDRAE
WP_081401608.1 MSKKLKCLTL FTAIFFAIPM AACQSLSQQK Q-----QLTT EKNDQQQISS LFQSAQTSGV LVIYDGKKIQ SYGNALDRAE
EPF93312.1 MSKKLKCLTL FTAIFFAIPM AACQSLSQQK Q-----QLTT EKNDQQQISS LFQSAQTSGV LVIYDGKKIQ SYGNALDRAE
OBY74151.1 MSKKLKCLTL FTAIFFAIPM AACQSLSQQK Q-----QLTT EKNDQQQISS LFQSAQTSGV LVIYDGKKIQ SYGNALDRAE
WP_023271137.1 MSKKLKCLTL FTAIFFAIPM AACQSFSQQK Q-----QLST QKNEQQQISS LFQSAQTSGV LVIYDGKKIQ SYGNDIHRAD
WP_005185807.1 MSKKLKMLTL SISMMLGLPL MACQSFSQQK Q-----QIMT QEREQQQITS LFQNAQTSGV LVIYDGKKIQ KFGNDVHRAD
WP_081401813.1 MSKKLKMLTL SISMMLGLPL MACQSFSQQK Q-----QIMT QESEQQQIAS LFQNAQTSGV LVIYDGKKIQ KFGNDVHRAD
WP_032877719.1 ---------- ---------- MACQSFSQQK Q-----QIMT QKSEQQQIAS LFQNAQTSGV LVIYDGKKIQ KYGNDTSRAE
WP_005297518.1 MSKRLKTLAL SASFTFALPL VACQSFGDQT Q-----HIMA QKSEQQNIAT LFQQAQTSGV LVIYDGKKIQ KYGNDTSRAE
G1
G13
G2
G12
G3
G4
G5
G6
G7
G8
G9
G10
G11

Accession	Sequence	Group
AVZ84486.1	TAYIPASTFK IANALIGLEN HKATSTEIFK WDGKPRFFKA WDKDFTLGEA MQASTVPVYQ ELARRIGPSL MQSELQRIGY	G1
WP_002002480.1	TAYIPASTFK IANALIGLEN HKATSTEIFK WDGKPRFFKA WDKDFTLGEA MQASTVPVYQ ELARRIGPSL MQSELQRIGY	
WP_002002480.1	TAYIPASTFK IANALIGLEN HKATSTEIFK WDGKPRFFKA WDKDFTLGEA MQASTVPVYQ ELARRIGPSL MQSELQRIGY	
AVZ84492.1	TAYIPASTFK IANALIGLEN HKATSTEIFK WDGKPRFFKA WDKDFTLGEA MQASTVPVYQ ELARRIGPSL MQSELQRIGY	
WP_002002480.1	TAYIPASTFK IANALIGLEN HKATSTEIFK WDGKPRFFKA WDKDFTLGEA MQASTVPVYQ ELARRIGPSL MQSELQRIGY	
WP_047430089.1	QRYIPASTFK MLNALIGIQH HKTSPNEVFK WDGQKRAFRS WEKDLTLAEA MQASAVPVYQ ELARRIGLEL MASEVKRVGY	G13
WP_005260134.1	QRYIPASTFK MLNALIGIQH HKTSPNEVFK WDGQKRAFRS WEKDLTLAEG MQASAVPVYQ ELARRIGLEL MASEVKRVGY	
WP_004776204.1	QRYIPASTFK MLNALIGIQH HKTSPNEVFK WDGQKRAFRS WEQDLTLAEA MQASAVPVYQ ELARRIGLEL MASEVKRVGY	
WP_062846262.1	QRYIPASTFK MLNALIGIQH HKTSPNEVFK WDGQKRAFRS WEKDLTLAEA MQASAVPVYQ ELARRIGLEL MASEVKRVGY	
WP_005282462.1	QRYIPASTFK MLNALIGIQH HKTSPNEVFK WDGQKRAFTS WEKDLTLAEA MQASAVPVYQ ELARRIGLEL MASEVKRVGY	G2
WP_005227858.1	QRYIPASTFK MLNALIGIQH HKTSPNEVFK WDGQKRVFTS WEKDLTLAEA MQASAVPVYQ ELARRIGLEL MASEVKRVGY	
WP_052209191.1	QRYVPASTFK ILNALIGLQY HKTTPDEVFK WDGQKRAFKQ WEKDLTLAQA MQVSAVPVYQ TLARRIGLQR MASEVKRVGY	G12
WP_081400730.1	TAYIPASTFK MLNALIGIQH HKTSPNEVFK WDGKKRAFAS WEKDLTLAEA MQASAVPVYQ ELARRIGLEL MANEVKRVGF	G3
WP_081398973.1	TAYIPASTFK MLNALIGIQH HKSSPNEVFK WDGKKRAFAS WEKDLTLAEA MQASAVPVYQ ELARRIGLEL MANEVKRVGF	
ENU35234.1	TAYIPASTFK MLNALIGIQH HKSSPNEVFK WDGKKRAFAS WEKDLTLAEA MQASAVPVYQ ELARRIGLEL MANEVKRVGF	G4
WP_004683508.1	TAYIPASTFK MLNALIGIQH HKSSPNEVFK WDGKKRAFAS WEKDLTLAEA MQASAVPVYQ ELARRIGLEL MANEVKRVGF	
ENU83658.1	TAYIPASTFK MLNALIGIQH HKSSPNEVFK WDGKKRAFAS WEKDLTLAEA MQASAVPVYQ ELARRIGLEL MANEVKRVGF	
ENU88055.1	TAYIPASTFK MLNALIGIQH HKSSPNEVFK WDGKKRAFAS WEKDLTLAEA MQASAVPVYQ ELARRIGLEL MANEVKRVGF	
ENU95367.1	TAYIPASTFK MLNALIGIQH HKSSPNEVFK WDGKKRAFAS WEKDLTLAEA MQASAVPVYQ ELARRIGLEL MANEVKRVGF	
ENX69881.1	TAYIPASTFK MLNALIGIQH HKSSPNEVFK WDGKKRAFAS WEKDLTLAEA MQASAVPVYQ ELARRIGLEL MANEVKRVGF	
ENU35234.1	TAYIPASTFK MLNALIGIQH HKSSPNEVFK WDGKKRAFAS WEKDLTLAEA MQASAVPVYQ ELARRIGLEL MANEVKRVGF	
WP_017402160.1	QRYIPASTFK MLNALIGIQH HKTTPDEMFK WDGKKRAFSS WEKDLTLAEA MQASAVPVYQ ELARRIGLEL MTREVKRVGY	G5
WP_005201559.1	QRYIPASTFK MLNALIGIQH HKTTPDEMFK WDGKKRAFSS WEKDLTLAEA MQASAVPVYQ ELARRIGLEL MTREVKRVGY	
WP_081402432.1	QRFIPASTFK ILNALIGIQH HKTTPNEVFK WDGQKRAFSS WEKDLSLAEA MQASAVPVYQ ELARRIGLEL MTREVKRVGY	G6
WP_081402432.1	QRFIPASTFK ILNALIGIQH HKTTPNEVFK WDGQKRAFSS WEKDLSLAEA MQASAVPVYQ ELARRIGLEL MTREVKRVGY	
WP_081402432.1	QRFIPASTFK ILNALIGIQH HKTTPNEVFK WDGQKRAFSS WEKDLSLAEA MQASAVPVYQ ELARRIGLEL MTREVKRVGY	
EPG42609.1	QRFIPASTFK ILNALIGIQH HKTTPNEVFK WDGQKRAFSS WEKDLSLAEA MQASAVPVYQ ELARRIGLEL MTREVKRVGY	
ENX33046.1	QRFIPASTFK ILNALIGIQH HKTTPNEVFK WDGQKRAFSS WEKDLSLAEA MQASAVPVYQ ELARRIGLEL MTREVKRVGY	
WP_026040241.1	QRYIPASTFK ILNALIGIQY HKTTPNEVFK WDGKKRTFSS WEKDLSLAEA MQASAVPVYQ ELARRIGLEL MTREVKRVGY	G7
WP_005238189.1	QRYIPASTFK ILNALIGIQY HKTTPNEVFK WDGKKRTFSS WEKDLSLAEA MQASAVPVYQ ELARRIGLEL MTREVKRVGY	
ENV10177.1	QRYIPASTFK MLNALIGIQH HKTTPDEVFK WDGKKRAFSS WEKDLTLAEA MQASAVPVYQ ELARRIGLEL MTREVKRVGY	G8
WP_005317413.1	QRYIPASTFK MLNALIGIQH HKTTPDEVFK WDGKKRAFSS WEKDLTLAEA MQASAVPVYQ ELARRIGLEL MTREVKRVGY	
WP_081406929.1	QRYIPASTFK MLNALIGIQH HKTTPDEVFK WDGKKRAFSS WEKDLTLAEA MQASAVPVYQ ELARRIGLEL MSREVKRVGY	
WP_016162389.1	QRYIPASTFK MLNALIGIQH HKTTPDEVFK WDGKKRAFSS WEKDLTLAEA MQASAVPVYQ ELARRIGLEL MTREVKRVGY	
WP_032812605.1	QRYIPASTFK MLNALIGIQH HKTTPDEVFK WDGKKRAFSS WEKDLTLAEA MQASAVPVYQ ELARRIGLEL MTREVKRVGY	
OEY96677.1	QRYIPASTFK MLNALIGIQH HKTTPDEVFK WDGKKRAFSS WEKDLTLAEA MQASAVPVYQ ELARRIGLEL MTREVKRVGY	
WP_081401608.1	QRYIPASTFK MLNALIGIQH HKTAPNEVFK WDGKKRAFSS WEKDLTLAEA MQASAVPVYQ ELARRIGLEL MTREVKRVGY	
EPF93312.1	QRYIPASTFK MLNALIGIQH HKTAPNEVFK WDGKKRAFSS WEKDLTLAEA MQASAVPVYQ ELARRIGLEL MTREVKRVGY	
OBY74151.1	QRYIPASTFK MLNALIGIQH HKTAPNEVFK WDGKKRAFSS WEKDLTLAEA MQASAVPVYQ ELARRIGLEL MTREVKRVGY	
WP_023271137.1	QRYIPASTFK MLNALIGIQH HKTTPDEVFK WDGKKRAFSS WEKDLTLAEA MQASAVPVYQ ELARRIGLEL MTREVKRVGY	
WP_005185807.1	QRYIPASTFK MLNALIGIQH HKTTPKEVFK WDGQKRAFSS WEKDLTLAEA MQASAVPVYQ ELARRIGLEL MTREVKRVGY	G9
WP_081401813.1	QRYIPASTFK MLNALIGIQH HKTTPKEVFK WDGQKRAFSS WEKDLTLAEA MQASAVPVYQ ELARRIGLEL MTREVKRVGY	
WP_032877719.1	HRYIPASTFK MLNALIGIQH HKTTPNEIFK WDGRKRAFSS WEKDLTLAEA MQASAVPVYQ ELARRIGLEL MTQEIKRVGY	G10
WP_005297518.1	QRYIPASTFK MLNALIGIQH HKTTPNEVFK WDGQKRAFSS WEKDLTLAEA MQASAVPVYQ ELARRIGLEL MTREVKRVGY	G11

	170	180	190	200	210	220	230	240	
AVZ84486.1	GNMQIGTEVD	QFWLKGPLTI	TPIQEVKFVY	DLAQGQLPFK	PEVQQQVKEM	LYVERRGENR	LYAKSGWGMA	VDPQVGWYVG	G1
WP_002002480.1	GNMQIGTEVD	QFWLKGPLTI	TPIQEVKFVY	DLAQGQLPFK	PEVQQQVKEM	LYVERRGENR	LYAKSGWGMA	VDPQVGWYVG	
WP_002002480.1	GNMQIGTEVD	QFWLKGPLTI	TPIQEVKFVY	DLAQGQLPFK	PEVQQQVKEM	LYVERRGENR	LYAKSGWGMA	VDPQVGWYVG	
AVZ84492.1	GNMQIGTEVD	QFWLKGPLTI	TPIQEVKFVY	DLAQGQLPFK	PEVQQQVKEM	LYVERRGENR	LYAKSGWGMA	VDPQVGWYVG	
WP_002002480.1	GNMQIGTEVD	QFWLKGPLTI	TPIQEVKFVY	DLAQGQLPFK	PEVQQQVKEM	LYVERRGENR	LYAKSGWGMA	VDPQVGWYVG	
WP_047430089.1	GNQNIGAQVD	NFWLVGPLEI	TPVEEVKFAY	ALAKQQLPFD	PSTQQQVRDM	LLIENVQGTR	IYAKSGWGMD	VHPQVGWWTG	G13
WP_005260134.1	GNQNIGAQVD	NFWLVGPLEI	TPVEEVKFAY	ALAKQQLPFD	PSTQQQVRDM	LLIENVQGTR	IYAKSGWGMD	VNPQVGWWTG	
WP_004776204.1	GNQNIGTQFD	NFWLVGPLEI	TPVEEVKFAY	ALAKQQLPFA	PSTQQQVRDM	LLIENVQGTR	IYAKSGWGMD	VNPQVGWWTG	
WP_062846262.1	GNQNIGTQVD	SFWLVGPLEI	TPVEEVKFAY	ALAKQQLPFD	PSTQQQVRDM	LLIENVQGIR	IYAKSGWGMD	VNPQVGWWTG	
WP_005282462.1	GNQSIGTQVD	NFWLVGPLEI	TPVEEVKFAY	ALAKKQLAFD	SSTQQQVKDM	LLIEDIQGTK	IYAKSGWGMD	VKPQVGWWTG	G2
WP_005227858.1	GNQSIGTQVD	NFWLVGPLEI	TPVEEVKFAY	ALAKKQLAFD	SSTQQQVKDM	LLIEDIQGTK	IYAKSGWGMD	VKPQVGWWTG	
WP_052209191.1	GNQSIGTQVD	NFWLVGPLEI	TPVEEVKFVY	ALAHQQLPFD	SLTQQQVKQM	LLIEDRNGTK	IYAKSGWGMD	VNPQVGWWTG	G12
WP_081400730.1	GNAEIGTQVD	DFWLVGPLKI	TPIEEVKFAY	ALANKQLEFD	QSVQKQVKQM	VFVDEVHGTK	IYAKSGWGMD	VTPQVGWWTG	G3
WP_081398973.1	GNTEIGTQVD	DFWLVGPLKI	TPVDEVKFAY	ALANKQLAFD	QSVQEQVKQM	VFVDEVHGTK	IYAKSGWGMD	VTPQVGWWTG	
ENU35234.1	GNAEIGTQVD	DFWLVGPLKI	TPVEEVKFAY	ALAHKQLTFD	QSVQEQVKQM	VLVDEVKGTK	IYAKSGWGMD	VTPQVGWWTG	G4
WP_004683508.1	GNAEIGTQVD	DFWLVGPLKI	TPVEEVKFAY	ALAHKQLTFD	QSVQEQVKQM	VLVDEVKGTK	IYAKSGWGMD	VTPQVGWWTG	
ENU83658.1	GNAEIGTQVD	DFWLVGPLKI	TPVEEVKFAY	ALAHKQLTFD	QSVQEQVKQM	VLVDEVKGTK	IYAKSGWGMD	VTPQVGWWTG	
ENU88055.1	GNAEIGTQVD	DFWLVGPLKI	TPVEEVKFAY	ALAHKQLTFD	QSVQEQVKQM	VLVDEVKGTK	IYAKSGWGMD	VTPQVGWWTG	
ENU95367.1	GNAEIGTQVD	DFWLVGPLKI	TPVEEVKFAY	ALAHKQLTFD	QSVQEQVKQM	VLVDEVKGTK	IYAKSGWGMD	VTPQVGWWTG	
ENX69881.1	GNAEIGTQVD	DFWLVGPLKI	TPVEEVKFAY	ALAHKQLTFD	QSVQEQVKQM	VLVDEVKGTK	IYAKSGWGMD	VTPQVGWWTG	
ENU35234.1	GNAEIGTQVD	DFWLVGPLKI	TPVEEVKFAY	ALAHKQLTFD	QSVQEQVKQM	VLVDEVKGTK	IYAKSGWGMD	VTPQVGWWTG	
WP_017402160.1	GNKNIGTQVD	NFWLVGPLKI	TPVEEVRFAY	ALAKQKLPFD	QSTQQQVKGM	LLIDEVQGTK	IYAKSGWGMD	VSPQVGWWTG	G5
WP_005201559.1	GNKNIGTQVD	NFWLVGPLKI	TPVEEVRFAY	ALAKQKLPFD	QSTQQQVKGM	LLIDEVQGTK	IYAKSGWGMD	VSPQVGWWTG	
WP_081402432.1	GNKNIGTQVD	NFWLVGPLKI	TPVEEVRFVY	ALAKQKLPFD	QSTQQQVKGM	LLVDEHQGTK	IYAKSGWGMD	VTPQVGWWTG	G6
WP_081402432.1	GNKNIGTQVD	NFWLVGPLKI	TPVEEVRFVY	ALAKQKLPFD	QSTQQQVKGM	LLVDEHQGTK	IYAKSGWGMD	VTPQVGWWTG	
WP_081402432.1	GNKNIGTQVD	NFWLVGPLKI	TPVEEVRFVY	ALAKQKLPFD	QSTQQQVKGM	LLVDEHQGTK	IYAKSGWGMD	VTPQVGWWTG	
EPG42609.1	GNKNIGTQVD	NFWLVGPLKI	TPVEEVRFVY	ALAKQKLPFD	QSTQQQVKGM	LLVDEHQGTK	IYAKSGWGMD	VTPQVGWWTG	
ENX33046.1	GNKHIGTQVD	NFWLVGPLKI	TPVEEVRFVY	ALAKQKLPFD	QSTQQQVKDM	LLVDEHQGTK	IYAKSGWGMD	VTPQVGWWTG	
WP_026040241.1	GNKNIGTQVD	NFWLVGPLQI	TPVEEVRFVY	ALAKQKLPFD	QSTQQQVKGM	LLADERQGTK	IYAKSGWGMD	VSPQVGWWTG	G7
WP_005238189.1	GNKNIGTQVD	NFWLVGPLQI	TPVEEVRFVY	ALAKQKLPFD	QSTQQQVKGM	LLADERQGTK	IYAKSGWGMD	VSPQVGWWTG	
ENV10177.1	GNKNIGTQVD	NFWLVGPLKI	TPIEEVRFAY	ALAKQKLPFD	QPTQQQVKAM	LLVDQIQGTK	IYAKSGWGMD	VSPQVGWWTG	G8
WP_005317413.1	GNKNIGTQVD	NFWLVGPLKI	TPIEEVRFAY	ALAKQKLPFD	QPTQQQVKAM	LLVDQIQGTK	IYAKSGWGMD	VSPQVGWWTG	
WP_081406929.1	GNKNIGTQVD	NFWLVGPLKI	TPVEEVRFAY	ALAKQKLPFD	QPTQQQVKAM	LLVDQIQGTK	IYAKSGWGMD	VSPQVGWWTG	
WP_016162389.1	GNKNIGTQVD	TFWLVGPLKI	TPVEEVRFAY	ALAKQKLPFD	QPTQQQVKAM	LLVDQIQGTK	IYAKSGWGMD	VSPQVGWLTG	
WP_032812605.1	GNKNIGTQVD	NFWLVGPLKI	TPVEEVRFAY	ALAKQKLPFD	QPTQQQVKAM	LLVDQIQGTK	IYAKSGWGMD	VSPQVGWWTG	
OEY96677.1	GNKNIGTQVD	NFWLVGPLKI	TPVEEVRFAY	ALAKQKLPFD	QPTQQQVKAM	LLVDQIQGTK	IYAKSGWGMD	VSPQVGWWTG	
WP_081401608.1	GNKNIGTQVD	NFWLVGPLKI	TPVEEVRFAY	ALAKQKLPFD	QPTQQQVKAM	LLVDQIQDTK	IYAKSGWGMD	VSPQVGWWTG	
EPF93312.1	GNKNIGTQVD	NFWLVGPLKI	TPVEEVRFAY	ALAKQKLPFD	QPTQQQVKAM	LLVDQIQDTK	IYAKSGWGMD	VSPQVGWWTG	
OBY74151.1	GNKNIGTQVD	NFWLVGPLKI	TPVEEVRFAY	ALAKQKLPFD	QPTQQQVKAM	LLVDQIQDTK	IYAKSGWGMD	VSPQVGWWTG	
WP_023271137.1	GNKNIGTQVD	NFWLVGPLKI	TPVEEVRFAY	ALAKQKLPFD	QPTQQQVKAM	LLVDQIQGTK	IYAKSGWGMD	VSPQVGWWTG	
WP_005185807.1	GNKNIGTQVD	NFWLVGPLKI	TPVEEVRFAY	ALAKQKLPFD	QSTQQQVKGM	LLVDEVHGTK	IYAKSGWGMD	VSPQVGWWTG	G9
WP_081401813.1	GNKNIGTQVD	NFWLVGPLKI	TPVEEVRFAY	ALAKQKLPFD	QSTQQQVKGM	LLIDEVQGTK	IYAKSGWGMD	VSPQVGWWTG	
WP_032877719.1	GNNNVGTQVD	NFWLVGPLKI	TPVEEVRFAY	ALAKQKLPFD	QSTQQQVKGM	LLLDEVQGAK	IYAKSGWGMD	VSPQVGWWTG	G10
WP_005297518.1	GNKNIGTQVD	NFWLVGPLKI	TPVEEVRFAY	ALAKQKLPFD	QSTQQQVKGM	LLIDEVQGTK	IYAKSGWGMD	VNPQVGWWTG	G11

```
                           250        260        270        280        290
                    ....|....| ....|....| ....|....| ....|....| ....|....| ....
AVZ84486.1          FVEKADGQVV AFALNMQMKA GDDIALRKQL SLDVLDKLGV FHYL------ ----   G1
WP_002002480.1      FVEKADGQVV AFALNMQMKA GDDIALRKQL SLDVLDKLGV FHYL------ ----
WP_002002480.1      FVEKADGQVV AFALNMQMKA GDDIALRKQL SLDVLDKLGV FHYL------ ----
AVZ84492.1          FVEKADGQVV AFALNMQMKA GDDIALRKQL SLDVLDKLGV FHYL------ ----
WP_002002480.1      FVEKADGQVV AFALNMQMKA GDDIALRKQL SLDVLDKLGV FHYL------ ----
WP_047430089.1      WVEQPNGQVT AFSLNMEMKK AEHADARKAI VYQALQQLGL LPQ------- ----   G13
WP_005260134.1      WIEQTNGQIT AFSLNMEMKK AEHADARKAI VYQALQQLGL LPQ------- ----
WP_004776204.1      WVEQPNGQIT AFSLNMEMKK AEHADARKAI VYQALQQLGL LPQ------- ----
WP_062846262.1      WVEQPNGQVT AFSLNMEMKK AEHADARKAI VYQALQQLGL LPQ------- ----
WP_005282462.1      WVEQPNGQVT AFSLNMEMKK AAHAEARKAI VYQALQQLGL LPQ------- ----   G2
WP_005227858.1      WVEQPNGQVT AFSLNMEMKK AAHAEARKAI VYQALQQLGL LPQ------- ----
WP_052209191.1      WVEQAGGQVT AFSLNMEIKK QQDLEARQLI VYQALQQLGG LPGQCLDKKR PSL-   G12
WP_081400730.1      WIEQPNGQVI AFSLNLEINK PEHGDARKAI VYQALQQLKL LQKQ------ ----   G3
WP_081398973.1      WIEQPNGQVI AFALNLEINK PEHGDARKAI VYQALQQLKL LQKQ------ ----
ENU35234.1          WIEQPNGQVI AFSLNMQINN SKQGDARKAI VYQALQQLKL LETQ------ ----   G4
WP_004683508.1      WIEQPNGQVI AFSLNMQINN SKQGDARKAI VYQALQQLKL LETQ------ ----
ENU83658.1          WIEQPNGQVI AFSLNMQINN SKQGDARKAI VYQALQQLKL LETQ------ ----
ENU88055.1          WIEQPNGQVI AFSLNMQINN SKQGDARKAI VYQALQQLKL LETQ------ ----
ENU95367.1          WIEQPNGQVI AFSLNMQINN SKQGDARKAI VYQALQQLKL LETQ------ ----
ENX69881.1          WIEQPNGQVI AFSLNMQINN SKQGDARKAI VYQALQQLKL LETQ------ ----
ENU35234.1          WIEQPNGQVI AFSLNMQINN SKQGDARKAI VYQALQQLKL LETQ------ ----
WP_017402160.1      WIEQANGKIT AFSLNMEMSR PEHAEARKAI VYQALQQLDL LAN------- ----   G5
WP_005201559.1      WIEQANGKIT AFSLNMEMSR PEHAEARKAI VYQALQQLDL LAN------- ----
WP_081402432.1      WIEQPNGKII AFSLNMQMSQ PEHADARKVI VYQALQELGL LAN------- ----   G6
WP_081402432.1      WIEQPNGKII AFSLNMQMSQ PEHADARKVI VYQALQELGL LAN------- ----
WP_081402432.1      WIEQPNGKII AFSLNMQMSQ PEHADARKVI VYQALQELGL LAN------- ----
EPG42609.1          WIEQPNGKII AFSLNMQMSQ PEHADARKVI VYQALQELGL LAN------- ----
ENX33046.1          WIEQPNGKII AFSLNMQMSQ PAHADARKVI VYQALQELGL LAN------- ----
WP_026040241.1      WIEQPNGKTI AFSLNMQMSQ PEHANARKVI VYQALQELGL LAN------- ----   G7
WP_005238189.1      WIEQPNGKTI AFSLNMQMSQ PEHANARKVI VYQALQELGL LAN------- ----
ENV10177.1          WIEQPNGKIT AFSLNMQMSQ PEHADARKVI VYQALQELGL LAH------- ----   G8
WP_005317413.1      WIEQPNGKIT AFSLNMQMSQ PEHADARKVI VYQALQELGL LAH------- ----
WP_081406929.1      WIEQPNGKIT AFSLNMQMSQ PEHADARKAI VYQALQELGL LAH------- ----
WP_016162389.1      WIEQPNGKIT AFSLNMQMSQ PEHADARKVI VYQALQQLGL LAH------- ----
WP_032812605.1      WIEQPNGKIT AFSLNMQMSQ PEHADARKAI VYQALQQLGL LAH------- ----
OEY96677.1          WIEQPNGKIT AFSLNMQMSQ PEHADARKAI VYQALQQLGL LAH------- ----
WP_081401608.1      WIEQPNGKVI AFSLNMQMSQ PEHADARKAI VYQALQQLGL LAT------- ----
EPF93312.1          WIEQPNGKVI AFSLNMQMSQ PEHADARKAI VYQALQQLGL LAT------- ----
OBY74151.1          WIEQPNGKVI AFSLNMQMSQ PEHADARKAI VYQALQQLGL LAT------- ----
WP_023271137.1      WIEQPNGKIT AFSLNMQMSQ PEHADARKAI VYQALQQLGL LAH------- ----
WP_005185807.1      WIEQANGKIT AFSLNMEMSR PEHTEARKAI VYQALQQLDL LAN------- ----   G9
WP_081401813.1      WIEQANGKVT AFSLNMEMSR PEHAEARKAI VYQALQQLDL LAN------- ----
WP_032877719.1      WIEQANGKIT AFSLNMEMSQ PEHAETRKAI VYQALQQLDL LVN------- ----   G10
WP_005297518.1      WIEQANGKVT AFSLNMEMNR PEHADARKAI VYQALQQLDL LAN------- ----   G11
```
