# Supplementary material for: Genetic and Phenotypic Features of a Novel Acinetobacter Species, Strain A47, Isolated From the Clinical Setting
Source: Front Microbiol. 2019 Jun 18;10:1375. doi: 10.3389/fmicb.2019.01375 (PMC6591377; doi:10.3389/fmicb.2019.01375)
Supplement: TABLE S4 — Predicted binding affinity between OXA-like from Acinetobacter sp. A47 and OXA-51 from A. baumannii against to doripenem, imipenem, and oxacillin. [file Table_4.DOCX]

**Supplementary Table 4**. Predicted binding affinity between OXA-like from *Acinetobacter* sp. A47 and OXA-51 from *A. baumannii* against to doripenem, imipenem, and oxacillin.

|  | Binding affinity (Kcal/mol) | | |
| --- | --- | --- | --- |
|  | Doripenem | Imipenem | Oxacillin |
| OXA-like - A47 | -10.90 | -9.74 | -10.15 |
| OXA-51- *A. baumannii* | -10.67 | -10.09 | -7.9 |
